# Clinical potential of PD-1/PD-L1 blockade therapy for renal cell carcinoma (RCC): a rapidly evolving strategy

**DOI:** 10.1186/s12935-022-02816-3

**Published:** 2022-12-12

**Authors:** Mohammadsaleh Jahangir, Omid Yazdani, Mohammad Saeed Kahrizi, Sara Soltanzadeh, Hamidreza Javididashtbayaz, Azam Mivefroshan, Saba Ilkhani, Romina Esbati

**Affiliations:** 1 Faculty of Medicine, Iran University of Medical Sciences, Tehran, Iran; 2 School of Medicine, Shahid Beheshti University of Medical Sciences, Tehran, Iran; 3 Department of Surgery, Alborz University of Medical Sciences, Karaj, Alborz Iran; 4 Department of Radiation Oncology, School of Medicine, Tehran University of Medical Sciences, Tehran, Iran; 5 Baran Oncology Clinic, Medical Faculty, Islamic Azad University of Mashhad, Mashhad, Iran; 6 Department of Adult Nephrology, Urmia University of Medical Sciences, Urmia, Iran; 7 Department of Surgery and Vascular Surgery, Shohada-ye-Tajrish Hospital, Shahid Beheshti University of Medical Science, Tehran, Iran

**Keywords:** Programmed death-1 (PD-1), Programmed death-ligand 1 (PD-L1), Renal cell carcinoma (RCC), Combination therapy, Resistance

## Abstract

Programmed death-1 (PD-1)/programmed death-ligand 1 (PD-L1) blockade therapy has become a game-changing therapeutic approach revolutionizing the treatment setting of human malignancies, such as renal cell carcinoma (RCC). Despite the remarkable clinical activity of anti-PD-1 or anti-PD-L1 monoclonal antibodies, only a small portion of patients exhibit a positive response to PD-1/PD-L1 blockade therapy, and the primary or acquired resistance might ultimately favor cancer development in patients with clinical responses. In light of this, recent reports have signified that the addition of other therapeutic modalities to PD-1/PD-L1 blockade therapy might improve clinical responses in advanced RCC patients. Until, combination therapy with PD-1/PD-L1 blockade therapy plus cytotoxic T lymphocyte antigen 4 (CTLA-4) inhibitor (ipilimumab) or various vascular endothelial growth factor receptors (VEGFRs) inhibitors axitinib, such as axitinib and cabozantinib, has been approved by the United States Food and Drug Administration (FDA) as first-line treatment for metastatic RCC. In the present review, we have focused on the therapeutic benefits of the PD-1/PD-L1 blockade therapy as a single agent or in combination with other conventional or innovative targeted therapies in RCC patients. We also offer a glimpse into the well-determined prognostic factor associated with the clinical response of RCC patients to PD-1/PD-L1 blockade therapy.

## Introduction

Renal cell carcinoma (RCC) denotes cancer resulting from the renal epithelium and includes about 90% of kidney cancers [1]. It includes > 10 histological and molecular subtypes, of which clear cell RCC (ccRCC) is the most common, yielding the most tumor-related deaths [2]. Localized RCC could be efficiently managed with surgery, while showing robust resistance to conventional chemotherapy by metastatic RCC [3, 4]. Nevertheless, groundbreaking advances in the treatment of metastatic RCC have been enabled with targeted compounds including axitinib, sunitinib, sorafenib, bevacizumab, everolimus, temsirolimus, cabozantinib, and pazopanib. They inhibit vascular endothelial growth factor (VEGF) and its receptor (VEGFR) or mechanistic target of rapamycin (mTOR) complex, eliciting an inhibitory effect on angiogenesis [5–7].

Currently, immunological analyses of RCC have caused important mechanistic and clinical perceptions. Indeed, immune infiltration properties of RCC are of growing interest by the increase of immune checkpoint inhibitor (ICI) therapy in this condition [8]. Notably, among 19 tumor types evaluated by The Cancer Genome Atlas (TCGA), a landmark cancer genomics program, RCC has the uppermost T cell infiltration score [9]. As well, advanced RCC has association with a rise in T helper 2 (Th2) and T regulatory cell (Tregs) infiltration [10]. These findings confer the importance of immunotherapy-based approaches to moderate RCC progress.

Immune checkpoints (ICs) denote specific membrane molecules situated mainly, but not exclusively, on T lymphocytes [11]. They bind responding ligands on antigen-presenting cells (APCs) like dendritic cells (DCs) or tumor cells [12]. Main cell surface inhibitory ICs encompass programmed cell death receptor-1 (PD-1 or CD279), cytotoxic T lymphocyte antigen-4 (CTLA-4), B and T lymphocyte attenuator (BTLA), lymphocyte activation-gene-3 (LAG-3) and T cell membrane protein-3 (TIM-3) [13, 14]. Apart from anti-angiogenic agents, more attention has been paid to immune checkpoint inhibitors (ICIs) such as nivolumab to manage metastatic RCC [15, 16]. Although conventional therapies such as chemotherapy and radiotherapy directly influence the tumor [17, 18], novel treatments such as the diversity of immunotherapies usually affect the microenvironment and the immune system. In fact, immunotherapeutics, such as ICIs, indirectly eliminate tumor cells through modifying the tumor microenvironment (TME) and/or effector immune cells [15, 19]. During the last decade, the ICIs targeting PD-1/PDL-1 interaction have shown promising results for the second-line treatment of metastatic RCC [20]. They establish apparent advantages such as broad applicability across cancer types and durable clinical response. Nonetheless, anti- PD-1/PDL-1 antibodies as a single agent remain ineffective in about 70–75% of RCC patients, especially in cancers with a low mutational burden [21, 22]. Hence, administration of dual ICI treatments or combining the PD1/PD-L1 blockade therapy with angiogenesis inhibitors and chemo-radiotherapy or other therapeutics might bypass RCC resistance to ICI therapy and also modify treatment-related adverse events (TRAEs) [23, 24].

Herein, we deliver an outline respecting the therapeutic capability of PD-1/PDL-L1 blockade therapy as a single agent or with other modalities for advanced RCC patients. Besides, a glimpse of the most applicable predictive biomarkers affecting a patient’s response to PD-1/PDL-L1 blockade therapy will be delivered.

## Immunotherapy for RCC

**Fig. 1:**
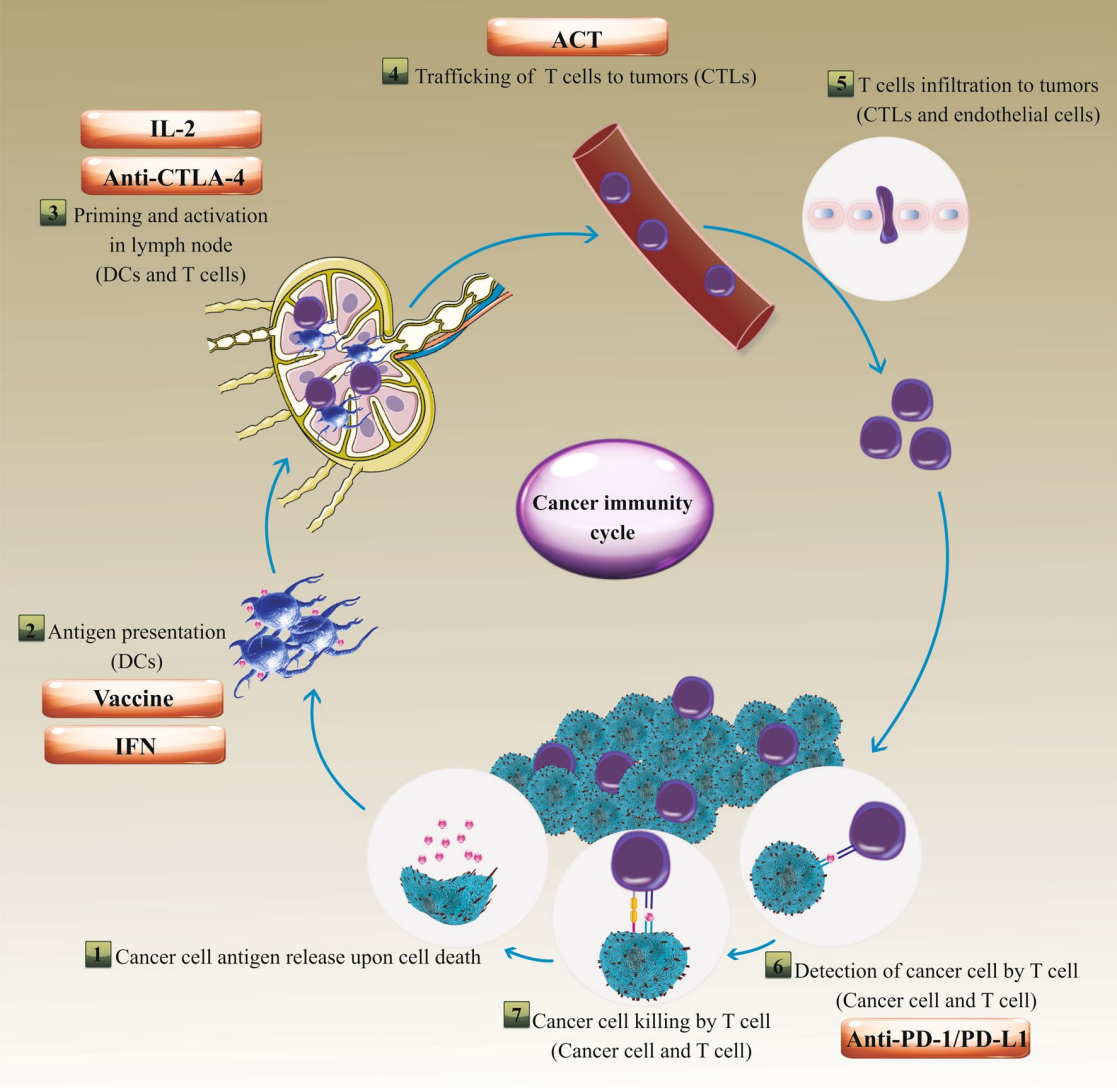
Various immunotherapy-based approaches for renal cell carcinoma (RCC) therapy

In principle, tumor progression can be regulated by cytotoxic innate and adaptive immune cells; however, as the tumor develops from neoplastic tissue to clinically detectable tumors, cancer cells evolve various mechanisms that mimic peripheral immune tolerance for deterring tumoricidal attack. Intrinsic mechanisms in cancer cells, including negative regulation of the major histocompatibility complex (MHC) class I and II molecules and/or tumor-associated antigens (TAAs) reduces presentation and resultant targeting by immune effector mechanisms [25, 26]. In addition to the secretion of immunosuppressive biomolecules like interleukin-10 (IL-10) and transforming growth factor-β (TGFβ), cancer cells also release immunosuppressive extracellular vesicles (EVs), in particular, exosomes [27–29]. Moreover, overexpressing PD-L1 and Fas ligand and tumor necrosis factor (TNF)-related apoptosis-inducing ligand (TRAIL) are other mechanisms by which tumor cells evade immune attack [30, 31]. Indeed, tumor-secreted molecules from the TME stimulate immunosuppression and thus restrict strong anti-tumor immune responses. Besides, tumor infiltration by tumor-associated macrophages (TAMs) and Tregs is intimately associated with weakened survival in RCC patients [32]. TAMs may inspire the tumor-infiltrating lymphocytes (TILs) towards a more regulated phenotype with a lower anti-tumor activity [32]. Accordingly, researchers have sought different tactics to detour tumor cell resistance to immune surveillance. Among them, more attention has been spent on the ICIs, anti-tumor cytokines (IL-2 and IFN-ɑ), cancer cell vaccine, and adoptive cell transfer (ACT) (Fig. 1).

### Immune checkpoint inhibitors

T cell-induced immunity includes various sequential steps encompassing the clonal selection of antigen-specific cells, their activation and growth in secondary lymphoid tissues, their trafficking to the regions of antigen and inflammation, the exerting of direct effector activity, and the provision of assistance (e.g., by cytokines and membrane ligands) for a diversity of effector immune cells [33]. Each noted step is fine-tuned by an equilibrium between stimulatory and inhibitory signals [34]. Almost all inhibitory signals in the immune response finally target intracellular signaling axes. These signals are predominantly transduced by membrane receptors, and their ligands are either membrane-bound or soluble (cytokines) [35, 36]. Universally, co-stimulatory and inhibitory receptors and ligands that tune T cell functions are not inevitably overexpressed in tumors relative to normal tissues. In contrast, inhibitory ligands and receptors that contribute to T cell effector activities in tissues are nearly overexpressed on transformed or non-transformed cells in the TME [37].

A myriad of ICs has been outlined and explored in cancer in past decades, encompassing but not restricted to PD-1, CTLA-4, LAG3, TIM3, T cell immunoreceptor with Ig and ITIM domains (TIGIT), and BTLA [38]. They are entitled to "immune checkpoints," denoting molecules that perform as gatekeepers of immune responses. The ICs shape various co-stimulatory and inhibitory interactions, causing self-tolerance and moderating physiological immune responses [39]. Also, they protect tissues from damage once the immune system is reacting to pathogenic infection and inhibits autoimmunity. Remarkably, tumors could deregulate their expression as a crucial immune resistance mechanism [40, 41]. In fact, ICs pathways, more importantly, PD-1/PD-L1 and CD28/CTLA-4, are co-opted by tumors, altering expression of proteins to ease cancer cells' evasion from immune surveillance as a result of the inhibiting T cell responses [14]. The ICs pathways mainly deter various key signaling axes in T cells, such as phosphatidylinositol 3-kinase (PI3K)/protein kinase B (AKT) and extracellular signal-regulated kinase 1/2 (ERK)/mitogen-activated protein kinase (MAPK), resulting in T cell exhaustion [42, 43]. Since the inhibitory activities of ICs are critically adjusted by their surface expression and signal transduction, targeting these axes offer efficient outcome in cancer patients [44]. The intravenous administration of ICIs, such as nivolumab, a human immunoglobulin G4 (IgG4) monoclonal antibody that binds to the PD-1 receptor, and ipilimumab, a fully human anti-CTLA-4 monoclonal antibody (IgG1), has gained approval from FDA as a first-line treatment of intermediate and poor-risk metastatic RCC with remarkable survival benefits across various clinical trials [45]. In contrast to most presently approved antibodies for cancer therapy, ICIs do not target cancer cells directly but rather target lymphocyte receptors or their ligands to favor endogenous anti-cancer activity.

### Cytokines

Cytokine-based immunotherapy is a promising approach in cancer therapy due to its competencies to moderate the host immune response toward the malignant cell and directly eliminate tumor cells [46, 47]. The history of utilizing cytokines as agents for the treatment of cancer initiated in the mid-1990s once the anti-cancer influences of high-dose (HD) IL-2 therapy was first displayed in RCC and other malignancies [48]. IL-2, as a well-known 15.5–16 kDa protein, boosts the cell-killing function of both natural killer (NK) cells and cytotoxic T cells (CTLs). IL-2 contributes to the maintenance of CD4 + regulatory T cells (Tregs) and the differentiation of CD4+ T cells into a variety of subsets [49, 50]. It also adjusts T-cell differentiation programs in response to antigen and enriches naive CD4+ T-cell differentiation into T helper-1 (Th1) and T helper-2 (Th2) cells while deterring T helper-17 (Th17) differentiation [51]. It was an early nominee for tumor immunotherapy and has been indicated for the treatment of metastatic RCC since 1992 and later for metastatic melanoma since 1998 [52, 53]. The HD IL-2 has an overall response rate (ORR) of 20% and a complete response rate (CR) of 8% in RCC patients [54]. Although various studies have assessed the effects of the IL-2 on the immune system leading to the evolvement of IL-2 application for tumor immunotherapy, several drawbacks hinder its utility. In this light, the dose and interval of the new IL-2-based reagents, the immunogenicity of the novel molecules, and their efficient combination have not yet been entirely clarified [55, 56].

Aside from the IL-2, IFN-α induces tumor regression in about 15% of patients with RCC. Studies indicate modest survival benefits for RCC patients treated with IFN-ɑ [57]. Importantly, the addition of the IL-2 to IFN-α results in a better CR but not OS in RCC patients [58]. Additions of other cytokines such as granulocyte–macrophage colony-stimulating factor (GM-CSF) to this combination may result in a marked increase in the number of peripheral blood mononuclear cells (PBMCs) expressing co-stimulatory molecules and thus might be valuable for immunotherapy of RCC [59]. There are 13 registered trials that are investigating the application of GM-CSF alone or plus other modalities in RCC patients. It was revealed that a higher dose of GM-CSF plus IL-2 led to superior T cell activation compared with a lower dose of IL-2 while exerting no effect on monocyte activation [60]. The combination therapy with IL-2 and IFN-α and chemo-radiotherapy may potentiate the anti-tumor capacity of IL-2 in RCC patients, as shown by improved ORR [61, 62]. Clinical benefits are positively associated with frameshift mutational load, mast cell tumor infiltration, reduced circulating tumor-associated T-cell clones, and T-cell clonal growth [61].

### Cancer vaccine

Therapeutic cancer vaccines have attracted growing attention in the last decade. The principal objective of the vaccine's application in cancer immunotherapy is the induction of the immune response toward transformed cells, bypassing the tolerance elicited by cancer [63, 64]. Nonetheless, not all kinds of tumors show an acceptable response to this modality. Vaccines are applied to treat slow-progressing immunogenic tumors that include specific tissue proteins. Recently, a more favored understanding of the extensiveness of tumor-associated antigens (TAAs), the native immunological response, and the progress of innovative machinery for antigen delivery have facilitated better vaccine design [65, 66]. The selection of the target antigen, as the most critical factor for advancing an anti-cancer vaccine, is of paramount importance because the massive majority of vaccines are intended to produce T-cell responses versus common TAA [67].

Cancer vaccines mainly include DNA, mRNA, peptide, protein, DCs, and tumor cell vaccines [68]. In 2010, the first cancer vaccine, sipuleucel-T (DC vaccine), gained approval from FDA to treat prostate cancer as a result of its capacity to prolong overall survival (OS) [69]. Among recently developed cancer vaccines, AGS-003 and IMA901 have displayed desirable outcomes in RCC patients [70, 71].

AGS-003, as an immunotherapeutic DC vaccine, has exhibited encouraging outcomes in combination with angiogenesis inhibitor sunitinib in phase II/III trials [72, 73]. The AGS-003 therapy aid in diminishing cancer-mediated impacts by presenting mature DC loaded with RNA to generate a more efficient and prolonged response [74]. When used plus sunitinib in a phase II study, IMA901 vaccines demonstrated significant clinical activity in RCC patients, as evidenced by improved OS [75]. IMA901 contains nine dissimilar human MHC-I binding-tumor-associated peptides and one MHC-II binding-tumor-associated peptide [76]. As a result, IMA901 induces an expansion of manifold T cells with diverse antigen specificities. Stimulating CD4+ and CD8+ T-cell responses toward TAA motivates a strong immune response, though specific against targets functionally relevant to tumor cells. In contrast, in a phase 3 trial, Rini and colleagues found that a combination of sunitinib with IMA901 did not ameliorate therapeutic outcomes in RCC patients [77]. The dissimilarity between the outcomes might be correlated to the action mechanism of vaccines because AGS-003 is made up of a reinforcement of APC, which enable T cells stimulation, and IMA901 is made up of small fragments of peptides expressed in cancer cells. Lacking the reinforcement of the APC eventually deters the efficacy of IMA901. Thus, the benefits of IMA901 might be authenticated in the prevention of recurrences.

The dendritic cell vaccine DC-Ad-GM.CAIX is an active, specific immunotherapy with efficient safety and efficacy against RCC [78]. It is a fusion-gene construct, granulocyte–macrophage (GM) colony-stimulating factor + CAIX, delivered by an adenoviral vector (Ad) into autologous dendritic cells (DCs). Recent published results from phase 1 trial (NCT01826877) showed that autologous immature DC-Ad-GM.CAIX can be safely used for metastatic RCC patients with no stern adverse events with CAIX-specific immune response induced by the treatment [79]. Cancer vaccine, in combination with other immunotherapy-based approaches such as cytokine therapy, in particular, low dose IL-2 and IFN-α, may stimulate promising outcomes in RCC patients [80, 81]. Of course, combining vaccines with cytokines provoke severe toxicity, as has been detected with IL-2 therapy. Further, the non-specific activation or growth of unwanted cell subsets, like Tregs, might induce global immunosuppression and thus limit vaccine responses. The low rate of clinical responses to combining cancer vaccines with IL-2, independent of dosing or schedule, indicates that IL-2 may not be an ideal option as an adjuvant [82]. We suggest that extension of IL-7 and IL-21 for the clinic provides the capacity to promote anti-tumor responses but with far less systemic toxicity without Treg proliferation.

### Adoptive cell transfer

Adoptive cell transfer (ACT) could be an effective treatment option in metastatic diseases, where conventional therapy inclines to fail [83, 84]. During ACT, immune cells (e.g., T cells and NK cells) are isolated from the patient or healthy donors, processed in vitro, extensively expanded, and finally administrated to the patient. Nonetheless, this modality has not been extensively exploited, mostly due to the small number of invasive lymphocytes and their inability to efficiently induce anti-tumor response [85]. Notably, the T or NK cell genetic engineering therapies can circumvent the drawbacks of low survival and migration of T cells and immune evasion [86, 87]. Until, complete regressions in patients with melanoma [88, 89] and lymphoma [90] have been ascertained utilizing the naturally TILs and anti-CD19-CAR-T cells, respectively. Before administration, patients can undergo lymphodepletion to attenuate the number of immune suppressor cells. In contrast to melanoma, ACT shows no remarkable clinical activity in other types of solid tumors like RCC [91]. Although TILs can be isolated from primary RCC samples, their immune response against RCC tumor cells is weak [92]. Also, the addition of the CD8+ TILs to low-dose IL-2 has no superiority over IL-2 alone in metastatic RCC patients. The ORR was 9.9% versus 11.4%, and the 1-year survival rate was 55% versus 47% in the TIL plus IL-2 arms and IL-2 alone arms, respectively [93]. Another phase I/II trial revealed that carboxy-anhydrase-IX (CAIX)-specific CAR T-cells elicited no clinical responses while inducing liver toxicity in RCC patients [94, 95].

Various other strategies are presently under examination; T cells are modified to target proteins expressed by RCC cells like MAGE-A3/12, CD70, DR4 and TRAIL, and ACT with autologous NK cells [96–98].

One of the facets that avert the effector action of the administrated TILs or engineered immune cells is the suppressive TME in RCC [99]. The tumor-stimulated immunosuppression is favored by producing the checkpoint receptor ligands accompanied by the secretion of anti-inflammatory cytokines such as IL-10 and TGF-β by the tumor cells. Such cytokines potentiate the cancer-supportive milieu, induce EMT, immune escape, and angiogenesis, and down-regulate apoptotic pathways [100]. Thus, much effort has been spent on developing other immunotherapy-based approaches, particularly ICIs.

## The rationality of targeting the PD-1/PD-L1 axis

Firstly, Ishida et al. discovered the PD-1 inhibitory receptor (CD279) in 1992 [101]. They suggested that PD-1 gene activation contributes to the classical type of programmed cell death [101]. In 1999, Nishimura et al. evinced its role in sustaining the peripheral immune tolerance by researching PD-1-deficient mice models [102]. Based on the literature, activated T cells, NK cells, B cells, macrophages, and DCs express the PD-1 on their surface [103–106]. The PD-1 expression on naïve T cells is prompted when TCR is activated [107]. This short-term expression is diminished in the absence of TCR signaling while increasing upon chronic activation, such as in chronic viral infections as well as tumors. The connection between PD-1 and its ligand, PD-L1, expressed on the cancer cell surface, barriers TCR signaling and CD28 co-stimulation and ultimately causes down-regulated T cell activity and ensuing tumor evasion [108].

The PD-1/PD-L1 interaction triggers signaling via the cytoplasmic tail of PD-1, resulting in T cell depletion. The PD-1 cytoplasmic tail consists of two tyrosine-based structural motifs, an immunoreceptor tyrosine-based inhibitory motif (ITIM) (V/L/I/XpYXX/L/V) and an immunoreceptor tyrosine-based switch motif (ITSM) (TXpYXXV/I) [109]. As a result of activation by PD-L1, the PD-1 phosphorylation occurs by Src kinases at ITIM and ITSM motifs. ITSM phosphotyrosine underlies the PD-1-mediated suppressive activities by recruiting Src homology region 2 domain-containing phosphatase-2 (SHP-2) [109, 110]. SHP-2 eliminates phosphate groups from neighboring effector proteins, in particular, PI3K and AKT, finally decreasing both cytokine manufacture and T cell growth [109]. Further, the nuclear factor kappa B (NF-κB) and mammalian target of rapamycin (mTOR) activation accompanied with the IL-2 and B-cell lymphoma-extra large (Bcl-xL) expression are decreased in activated T cells following PD-1/PD-L1 interaction. These events, in turn, inhibit T cells proliferation, cytotoxicity, and cytokine release, promote the apoptosis of tumor-specific T cells, up-regulate the differentiation of CD4+ T cells into foxp3+ Tregs, and finally potentiates tumor cell's resistance to CTL attack [111, 112]. In the lack of PD-1 signaling, number of long-lived plasma cells was evidently decreased [113]. Improved expression of PD-L1 is usually found in cancers and correlates with metastatic disease stage and undesired prognosis in RCC, gastric cancer, melanoma, breast cancer and etc. [114–116]. Iacovelli et al. (2016) demonstrated that PD-L1 was expressed in 24.2% of RCC tumors, and a higher level of PD-L1 expression augmented the risk of death by 81% [117]. Another study on 1,644 patients also signified the association between PD-L1 expression and OS in RCC patients [118]. Thus, scientists have pursued varied tactics to prohibit PD-1/PD-L1-mediated inhibitory impacts on T cells [119]. As described, negative regulation of this pathway employing PD-1-or PD-L1-targeting anti-bodies has become a promising plan with preferred clinical responses in numerous solid tumors like RCC [120, 121].

## Anti-PD-1 antibody in RCC patients

### Nivolumab

Nivolumab (OPDIVO®), a fully human IgG4 targeting PD-1, has boosted OS in RCC patients [122]. It was developed under a research collaboration entered in 2005 between Ono and Medarex. As a single agent or in combination with ipilimumab (anti-CTLA-4 antibody) or cabozantinib (anti-angiogenic agent), nivolumab has been approved to treat RCC patients [123–125].

#### Monotherapy

Results from CheckMate 025 study demonstrated that the OS was longer and high-grade adverse events were lower in RCC patients who underwent nivolumab than everolimus, an mTOR inhibitor [126–128]. CheckMate 025 study was conducted on 821 patients and exhibited that intravenous administration of nivolumab 3 mg/kg every 2 weeks increased OS to 25.0 months compared with 19.6 months in the everolimus arm [126]. The ORR was also more remarkable in nivolumab than in the everolimus arm (25% versus 5%) without significant differences in PFS [126, 129]. Based on the CheckMate 025 study results, nivolumab monotherapy was approved by the FDA in 2015 as a second-line treatment for RCC patients who have received prior anti‐angiogenic therapy. Another trial suggested that liver metastases and central nervous system (CNS) metastases at diagnosis were associated with worse OS, while pancreatic metastases at diagnosis were correlated to a better prognosis in RCC patients undergoing nivolumab therapy [130]. CheckMate 025 study also exhibited that the most common nivolumab treatment-related adverse events (TRAEs) were fatigue (34.7%) and pruritus (15.5%) in previously treated RCC [129]. Similarly, phase IIIb/IV CheckMate 374 study (NCT02596035) documented the clinical activity of nivolumab monotherapy 240 mg every 2 weeks in previously treated RCC, as shown by median OS of about 21.8 months [131]. In contrast to cited trials, nivolumab shows no superiority over angiogenesis inhibitor axitinib, an FDA-approved tyrosine kinase inhibitor (TKI), in RCC [132]. A clinical trial on 80 patients indicated that the clinical benefit rate of axitinib was meaningfully higher than that of nivolumab, with no difference in the OS of the two groups [132]. Also, baseline neutrophil-to-lymphocyte ratio (NLR), a marker of systemic inflammation, was associated with PFS, making it evident that NLR may be an efficient prognostic factor in RCC patients treated with nivolumab [132].

#### Combination therapy

In addition to monotherapy, nivolumab plus ipilimumab or TKI cabozantinib has been approved by FDA for RCC [45, 133]. A phase I CheckMate 016 study showed that intravenous nivolumab 3 mg/kg plus ipilimumab 1–3 mg/kg led to manageable safety, robust anti-cancer activity, and durable responses with promising OS in patients with metastatic RCC [134]. These preliminary results were authenticated by phase 3 CheckMate 214 study, where Motzer et al. (2018) displayed that intravenous administration of nivolumab 3 mg/kg every 2 weeks plus ipilimumab 1 mg/kg every 3 weeks improved OS and PFS and indicated better ORR compared sunitinib therapy (50 mg daily for 4 weeks) in RCC patients [45]. Based on the results of this study, this regimen was approved for RCC in 2018. Nivolumab plus ipilimumab has also been approved for BRAF V600 wild-type and BRAF V600 mutation-positive unresectable or metastatic melanoma (CheckMate 067), urothelial carcinoma (UC) (CheckMate 901), HCC (CheckMate 040) and also non-small cell lung cancer (NSCLC) with PD-L1 tumor expression ≥ 1% (CheckMate 227).

Recently, Choueiri et al. have assessed the safety and efficacy of nivolumab plus anti-angiogenic drug cabozantinib versus TKI sunitinib in the treatment of previously untreated RCC (CheckMate 9ER) [124, 135]. The phase 3 CheckMate 9ER study exhibited that nivolumab (240 mg intravenously every 2 weeks) plus cabozantinib (40 mg orally once daily) resulted in better ORR compared to sunitinib (50 mg once daily for 4 weeks) (55.7% versus 27.1%) in RCC patients [124, 136]. Further, the probability of OS at 1-year was 85.7% versus 75.6% in nivolumab plus cabozantinib arms compared to sunitinib arms. The results of this study verified the substantial benefits of nivolumab plus cabozantinib over sunitinib concerning the PFS and OS factors in previously untreated RCC [124, 137]. These consequences led to the approving nivolumab plus cabozantinib as first-line treatment for advanced RCC in 2021. Contrariwise, the addition of standard doses of anti-angiogenic drugs sunitinib or pazopanib to nivolumab caused a high occurrence of high-grade toxicities, restricting future progress of either combination regimen [138]. Hence, it seems that the success of combination regimens based on nivolumab and anti-angiogenic agents may be dependent on careful selection of the anti-angiogenic drugs as well as dose. Further studies also are investigating to address the safety and efficacy of combination therapy with nivolumab and other modalities in RCC [139–141]. In this light, Choueiri et al. (2021) showed that orally administration of C-X-C chemokine receptor type 4 (CXCR-4) inhibitor mavorixafor (400 mg daily) improved the clinical activity of nivolumab mainly by a reduction in the recruitment of immunosuppressive cells into the TME and enhancement in activated CTL infiltration [139]. Also, the addition of the bempegaldesleukin, a PEGylated IL-2, to nivolumab brought about a fortunate outcome irrespective of baseline PD-L1 status and baseline levels of TILs, indicating a therapeutic capacity for participants with poor prognostic risk factors for response to PD-1/PD-L1 inhibitors [140]. On the other hand, there is evidence suggesting that CBM588 could improve the clinical outcome in RCC patients treated with nivolumab plus ipilimumab [141]. The CBM588, as a bifidogenic live bacterial product, improves response to checkpoint inhibitors (CPIs) by affecting the gut microbiome [142, 143]. Notably, Dizman et al. showed that levels of IL-1β, G-CSF, IL-10, IL-12, GM-CSF, macrophage inflammatory protein-β (MIP-β), monocyte chemoattractant protein-1 (MCP-1), IL-1 receptor antagonist (IL-1RA), TNF-α, IL-2, IFN-γ-inducible protein 10 (IP-10), IL-2 receptor (IL-2R) and IL-8 increased in patients treated with nivolumab and ipilimumab plus CBM588 arm compared with nivolumab plus ipilimumab arm [141]. Thus, it appears that triggering an immune response by this product could potentiate immune response induction and subsequently heightens ICIs clinical activity. Notwithstanding, larger trials are needed to prove this clinical observation and clarify the action mechanism and the effects on the microbiome and immune compartments.

### Pembrolizumab

Pembrolizumab (KEYTRUDA), a humanized IgG4 monoclonal antibody targeting PD-1, has been approved as a single agent for the adjuvant treatment of patients with RCC at intermediate-high or high risk of recurrence following nephrectomy, or following nephrectomy and resection of metastatic lesions [144]. It was invented by scientists at Organon in cooperation with Medical Research Council Technology (MRCT). It also is indicated in combination with axitinib or lenvatinib, two well-known angiogenesis inhibitors, as the first-line treatment for patients suffering from advanced RCC [145, 146].

#### Monotherapy

Recently, a double-blind, phase 3 trial KEYNOTE-564 study exhibited that intravenous administration of pembrolizumab (at a dose of 200 mg) enhanced OS in clear-cell RCC patients who were at high risk for recurrence after nephrectomy [147, 148]. This trial was funded by Merck Sharp and Dohme and conducted on 496 patients. Results demonstrated that pembrolizumab therapy led to a longer disease-free survival (DFS) compared to the control group [147, 149, 150]. Also, the severe adverse events happened in 32.4% of the patients in the pembrolizumab arm versus 17.7% in the control arm, with no treatment-related deaths [147, 149, 150]. Based on the results of the KEYNOTE-564 study, pembrolizumab was approved for RCC therapy in 2021. In terms of safety profile, grade 3–5 treatment-related adverse events (TRAEs), in particular colitis and diarrhea, were shown in 30% of pembrolizumab-treated patients [151]. In addition to the clear cell RCC, pembrolizumab monotherapy exhibited promising anti-tumor activity in non-clear cell RCC, as shown by results from KEYNOTE-427 study [152]. In this trial, administration of the pembrolizumab 200 mg intravenously once every 3 weeks resulted in an ORR of about 26.7%, with a duration of response of 29.0 months [152]. Also, PFS and OS were 4.2 months and 28.9, respectively, signifying the promising clinical activity of first-line pembrolizumab monotherapy in non-clear cell RCC [152]. Nonetheless, these results must be validated in phase 3 trials prior to being approved by FDA.

#### Combination therapy

A myriad of studies have tested the safety and efficacy of combination therapy with pembrolizumab plus other treatments, most importantly, anti-angiogenic drugs (e.g., axitinib and lenvatinib) for RCC therapy [153, 154]. Since 2019, pembrolizumab plus axitinib has been indicated for the first-line treatment of patients with advanced RCC. The approval was based on the data from KEYNOTE 426 study on 861 patients with stage IV clear-cell RCC [155]. This phase 3 trial was conducted between Oct 24, 2016, and Jan 24, 2018, and exhibited that pembrolizumab (200 mg every 3 weeks intravenously) plus axitinib (5 mg orally twice daily) might elicit superior clinical outcomes over sunitinib (50 mg once daily), as evinced by longer OS and PFS [155]. Hypertension, which was detected in 22% of patients, was the most common grade 3–5 side effect of the combination regimen [155]. Other reports showed that the most common potential adverse event related to this regimen was diarrhea (29%) [156]. These findings validated the manageable safety profile of this regimen. However, incremental cost-effectiveness ratios of pembrolizumab in combination with axitinib versus sunitinib are $249,704 versus $150,000 per quality-adjusted life-year [157]. Hence, this combination regimen is not cost-effective versus sunitinib as a first-line treatment for patients with advanced RCC. In addition to the axitinib, the addition of the lenvatinib to pembrolizumab caused significant clinical activity in advanced RCC patients [146]. Accordingly, results from the CLEAR study exhibited that lenvatinib (20 mg orally once daily) in combination with pembrolizumab (200 mg intravenously once every 3 weeks) correlated with significantly longer PFS and OS than sunitinib (50 mg orally once daily) [146]. In August 2021, the CLEAR study results led to approving the combination of lenvatinib plus pembrolizumab for the first-line treatment of advanced RCC. Pembrolizumab with lenvatinib also has gained approval from FDA for endometrial cancer patients based on data from the KEYNOTE-775 study [158]. In contrast to the promising outcome, addition of the other types of anti-angiogenic agents like pazopanib to pembrolizumab might result in significant hepatotoxicity [159]. Thereby, careful consideration must be taken concerning the anti-angiogenic agent types and study design. Besides, the addition of the Pegilodecakin (pegylated recombinant human IL-10) [160] or pegylated IFNα-2b (PEG-IFN) [161] to pembrolizumab demonstrated a manageable toxicity profile and preliminary anti-cancer activity in RCC patients mainly by provoking the CTLs anti-cancer activities.

### Sintilimab

Sintilimab (Tyvyt), a fully human IgG4 monoclonal antibody, is an investigational PD-1 inhibitor developed by Innovent and Lilly [162]. Sintilimab has recently shown promising outcomes with durable response in lymphoma but not in solid tumors [163, 164]. It was recently approved in China for the treatment of classical Hodgkin’s lymphoma (cHL) and is undergoing phase I and II development to utilize in several solid tumors, including NSCLC and RCC. Safety and efficacy of sintilimab monotherapy (100 mg, 200 mg intravenously, once every three weeks) are investigated in fumarate hydratase (FH)-deficient RCC in phase 1/2 trials (NCT04146831 and NCT04387500). Besides, combination therapy with sintilimab and axitinib showed clinical activity in intermediate- and high-risk advanced RCC [165]. Meanwhile, combination therapy with sintilimab 200 mg intravenously every 3 weeks and axitinib 5 mg orally twice daily led to ORR of about 40% and the disease control rate (DCR) of about 90% with tolerable adverse effects in RCC patients [165]. Likewise, the addition of the pazopanib to 6–8 cycles of sintilimab improved PFS in RCC patients [166]. Because of the severe and fatal hepatotoxicity resulting from pazopanib therapy observed in RCC, monitoring hepatic function is urgently required during administration [167]. Further, combination therapy with sintilimab and chemotherapy (gemcitabine, oxaliplatin, capecitabine, irinotecan, nab-paclitaxel, tegafur, or nedaplatin) or sintilimab plus other anti-angiogenic agents (anlotinib or sorafenib) showed acceptable safety with rises in the treatment efficacy and DCR for advanced tumors like RCC [168]. It should be noted that due to the few adverse reactions and proven efficacy, sintilimab combination therapy can be applied as a potent strategy for the treatment of advanced RCC.

A brief overview of clinical trials targeting PD-1 alone or in combination with other treatments in RCC patients has been delivered in Table 1.

**Table 1:** Anti-PD-1 antibody alone or in combination with other treatments in RCC patients

Agents	Phase	Participants	Dose	Outcome	Refs.
Nivolumab Ipilimumab	3	1096	3 mg/kg 1 mg/kg	Better ORR and OS in nivolumab plus ipilimumab arm compared to sunitinib arm (ORR about 75% versus 60%)	[45]
Nivolumab Cabozantinib	3	651	240 mg 40 mg	Better PFS and OS (18.1 months versus 8.3 months) in nivolumab plus ipilimumab arm compared to sunitinib arm	[124]
Nivolumab	3	821	3 mg/kg	Improved OS (25.0 months versus 19.6 months) with lower serious adverse event in nivolumab arm compared to everolimus arm	[22]
Nivolumab Ipilimumab	3	847	3 mg/kg 1 mg/kg	Better patient-reported outcomes (PROs) in dual ICI arms than in sunitinib arm	[212]
Nivolumab Ipilimumab	1	6	3 mg/kg 1 mg/kg	Manageable safety, durable responses with promising OS	[134]
Nivolumab Ipilimumab CBM588	2	30	3 mg/kg 1 mg/kg 80 mg	Better PFS (12.7 months versus 2.5 months) in dual ICI plus CBM588 compared to nivolumab plus ipilimamab	[141]
Nivolumab Cabozantinib	3	323	240 mg 40 mg	Better PROs in nivolumab plus cabozantinib arm versus sunitinib arm	[213]
Nivolumab	2	720	3 mg/kg	Limited clinical activity	[214]
Nivolumab APX005M Cabiralizumab	1	26	240 mg 0.3 mg/kg 4 mg/kg	Acceptable safety and pharmacodynamic activity	[215]
Nivolumab	3	821	3 mg/kg 10 mg	High levels of CD8 + TILs expressing PD-1 might be a prognostic factor of response to anti-PD-1	[216]
Nivolumab Mavorixafor	1	9	240 mg 400 mg	Anti-activity and a manageable safety profile	[139]
Nivolumab	4	97	240 mg	Significant ORR (22.7%)	[217]
Nivolumab	–	80	240 mg	Axitinib had superiority over nivolumab in terms of PFS (10.3 months versus 7.3 months)	[132]
Nivolumab	2	73	3 mg/kg	Limited anti-tumor activity	[218]
Nivolumab Bempegaldesleukin	1/2	49	360 mg 0.006 mg/kg	Preliminary anti-tumor activity with the manageable safety profile	[219]
Nivolumab RT	2	69	240 mg 10 Gy	No significant clinical activity	[220]
Nivolumab Sunitinib Pazopanib	1	33	2 mg/kg 50 mg 800 mg	Higher rates of high-grade toxicities	[138]
Pembrolizumab Axitinib	3	861	200 mg 5 mg	Better OS, PFS, and ORR in the combination arm versus sunitinib arm	[145]
Pembrolizumab Lenvatinib	3	1069	200 mg 20 mg	Longer PFS ( 23.9 versus 9.2 months) and OS in the combination arm versus sunitinib arm	[146]
Pembrolizumab Lenvatinib	1b/2	137	200 mg 20 mg	Manageable safety profile and promising clinical activity	[221]
Pembrolizumab	2	165	200 mg	Significant ORR (26.7%) along with remarkable PFS (4.2 months) and OS (28.9 months)	[152]
Pembrolizumab Axitinib	1b	11	2 mg/kg 5 mg	The intervention was well tolerated and exhibited clinical activity	[156]
Pembrolizumab Bevacizumab	1b/2	48	200 mg 15 mg/kg	The combination regimen was safe and active	[222]
Pembrolizumab Axitinib	1	-	2 mg/kg 5 mg	Immune-related biomarkers had an intimate association with better ORR and PFS	[223]
Pembrolizumab RT	1/2	30	200 mg 20 Gy	Robust clinical activity (ORR: 63% and DCR: 83%)	[224]
Pembrolizumab Pegylated IFNɑ-2b or Ipilimumab	1b	22	2 mg/kg 2 μg/kg 1 mg/kg	The combination regimen was safe and active	[161]
Toripalimab	1	6	10 mg/kg	Acceptable clinical activity	[225]

Programmed cell death protein 1 (PD-1), Renal cell carcinoma (RCC), Overall survival (OS), Objective response rate (ORR), Progression-free survival (PFS), Disease control rate (DCR), Tumor-infiltrating lymphocytes (TILs), Radiotherapy (RT)

## Anti-PD-L1 antibody in RCC patients

### Atezolizumab

Atezolizumab (Tecentriq) is a fully-humanized, engineered IgG1 monoclonal antibody against the PD-L1 [169]. It firstly was developed by Genentech/Roche and has recently been approved to treat urothelial carcinoma (UC) [170], lung cancer [171, 172], triple-negative breast cancer (TNBC) (in combination with paclitaxel) [173], and hepatocellular carcinoma (HCC) (in combination with anti-angiogenic drugs) [174]. Also, atezolizumab shows significant clinical activity against RCC [175, 176]. For some cancers, PD-L1 expression levels are suspected as a prognostic factor, but most cancers with PD-L1 expression still do not respond.

#### Monotherapy

In 2016, a phase 1 trial in 70 patients with metastatic RCC showed that intravenously every 3 weeks administration of atezolizumab (0.01, 0.03, 0.1, 0.3, 1, 3, 10, 20 mg/kg) has a manageable safety profile and might elicited objective response [177]. Grade 3 TRAEs happened in 17% of patients, with no grade 4 or 5 events. Also, the treatment improved the OS and PFS of RCC patients to 28.9 months and 5.6 months, respectively [177]. Study of the possible prognostic factors signified that a drop in circulating plasma markers and acute-phase proteins in combination with elevated baseline effector T-cell-to-Tregs gene expression ratio has a correlation with response to atezolizumab [177]. For the first time, this study showed the safety and clinical activity of atezolizumab in RCC. Regardless of this report, there is no reliable report to verify the clinical activity of atezolizumab for RCC. Meanwhile, a phase 3 trial (IMmotion010) is ongoing to evaluate the efficacy of atezolizumab as adjuvant therapy in RCC patients at high risk of developing metastasis following nephrectomy [178, 179].

#### Combination therapy

In the last years, researchers have sought various approaches to potentiate the efficacy and ameliorate the safety profile of atezolizumab in RCC patients. In this regard, anti-angiogenic drugs, in particular bevacizumab and cabozantinib, have attracted growing attention [6, 180]. A randomized phase 2 IMmotion150 study provides clear evidence that combination therapy with atezolizumab and bevacizumab has superiority over sunitinib in terms of the PFS [181]. Interestingly, biomarker analyses showed that tumor mutation burden (TMB) and neoantigen burden has no association with PFS. It was thus suggested that blocking VEGF by bevacizumab may defeat resistance to atezolizumab [179, 181]. After that, a phase 3 trial IMmotion151 study indicated that atezolizumab 1200 mg plus bevacizumab 15 mg/kg intravenously once every 3 weeks improved PFS more evidently than sunitinib (11.2 months versus 7.7 months) in RCC patients with better safety profile [175]. These results authenticated the clinical activity of atezolizumab plus bevacizumab as a first-line treatment for RCC. Notwithstanding, the final analysis of phase 3 IMmotion151 trial displayed no significant enhancement in OS with atezolizumab plus bevacizumab over sunitinib for previously untreated RCC patients [182, 183]. As a result, FDA has not yet approved this treatment regimen for RCC, while atezolizumab plus bevacizumab has previously been approved for other tumors, such as HCC [174]. Besides, another trial showed that the addition of the PEG-IFNα-2a to atezolizumab might have preliminary clinical activity and acceptable tolerability in advanced RCC patients [184]. As described, IFN-α improves tumor immunogenicity and DC response to the tumor, augments Th1/Th2 ratio, and thus potentiates T cell-mediated cytotoxicity [185, 186]. Although IFN-α plus bevacizumab has been approved for RCC [187, 188], its clinical activity when used plus atezolizumab is being investigated and has not yet strongly been documented. Further, Jung et al. reports (2019) for the first time signified that the addition of indoleamine 2,3-dioxygenase 1 (IDO1) inhibitor, navoximod, to atezolizumab might improve its efficacy in advanced tumors like RCC [189]. IDO1 triggers immune suppression in T cells by l-tryptophan depletion and kynurenine collections in the TME, suppressing CTL and Th1 cells and promoting Tregs activity [190, 191]. Thus, targeting its activity may be a rational strategy to alleviate tumor progress [192, 193]. Achieved results implied that a combination of navoximod and atezolizumab had acceptable safety, tolerability, and pharmacokinetics for patients with advanced tumors such as RCC [189]. Nonetheless, further information is required to corroborate the benefit of adding navoximod to atezolizumab.

### Avelumab

Avelumab (Bavencio®) is an IgG1 mAb directed to PD-L1 that was discovered by Merck KGaA and Pfizer [194]. As a single agent, it has been approved for the metastatic Merkel cell carcinoma (MCC) [195] and also UC [196]. FDA also approved avelumab in combination with axitinib for the first-line treatment of advanced RCC patients [197].

#### Monotherapy

Study of the safety and efficacy and avelumab monotherapy in patients with advanced RCC verified its clinical activity in a phase 1 trial [198]. Meanwhile, avelumab 10 mg/kg intravenously every 2 weeks led to an ORR of about 16.1%, with median DOR and PFS about 9.9 and months 8.3, respectively [198]. Also, the intervention showed a manageable safety profile [198]. Nonetheless, there was no further proof showing the clinical activity of avelumab as a single agent in phase 2/3 trials. As described, avelumab monotherapy is indicated for UC and MCC based on results from phase 3 JAVELIN Bladder 100 and JAVELIN Merkel 200 study, respectively. It’s acceptable safety profile and capability to stimulate durable responses in otherwise deadly tumors offer the justification for its application in other tumor types and in combination with other therapeutic approaches.

#### Combination therapy

Recently, a phase 3 JAVELIN Renal 101 study on 886 RCC patients exhibited that the addition of the axitinib to avelumab caused objective responses in patients with advanced RCC [199, 200]. Motzer et al. showed that avelumab 10 mg/kg intravenously every 2 weeks plus axitinib 5 mg orally twice daily had superiority over sunitinib 50 mg orally once daily for 4 weeks in terms of ORR and PFS [199]. The median PFS in PD-L1-positive tumors was 13.8 months in the combination therapy arm compared with 7.2 months in the sunitinib arm [199, 201]. In the same population, ORR was 55.2% versus 25.5% in the combination therapy arm compared with the sunitinib arm. These results suggested this regimen as a first-line treatment for advanced RCC [199, 201]. In May 2019, based on the JAVELIN Renal 101 study results, the FDA approved avelumab in combination with axitinib for the first-line treatment of people with advanced RCC. Study of the possible prognostic factor presented NLR as a prognostic biomarker in advanced RCC patients who underwent avelumab plus axitinib or sunitinib administration [202]. There was an association with baseline NLR and OS, and PFS in advanced RCC patients who received avelumab plus axitinib [202]. Accordingly, patients with below-median NLR experienced extended PFS and OS. Interestingly, median PFS was 13.8 and 11.2 months in RCC patients with below-median NLR and 13.3 and 5.6 months in patients with median-or-higher NLR [202]. These analyses confer the role of NLR in underlying mechanisms affecting clinical outcomes.

A brief overview of clinical trials targeting PD-L1 alone or in combination with other treatments in RCC patients has been delivered in Table 2.

**Table 2:** Anti-PD-L1 antibody alone or in combination with other treatments in RCC patients

Agents	Phase	Participants	Dose	Outcome	Refs.
Atezolizumab Bevacizumab	2	305	1200 mg 15 mg/kg	Improved PFS, which had no association with tumor mutation and neoantigen burden	[179]
Atezolizumab Bevacizumab	3	915	1200 mg 15 mg/kg	Improved PFS versus sunitinib (11·2 months versus 7.7 months) with a favorable safety profile	[175]
Atezolizumab Cabozantinib	1b	102	1200 mg 40–60 mg	Prolonged PFS to19.5 months	[226]
Atezolizumab Bevacizumab	2	59	1200 mg 15 mg/kg	Improved PFS (8.7 moths) with detection of TRAEs in 83% of patients	[227]
Atezolizumab Interferon-α	1b	158	1200 mg 180 μg	Significant ORR (20.0%)	[184]
Atezolizumab	1	17	0.01–20 mg/kg	Improved OS (28.9 months) and PFS (5.6 months)	[177]
Atezolizumab Navoximod	1	157	50–1000 mg	Acceptable safety, tolerability, and pharmacokinetics	[228]
Avelumab Axitinib	3	886	10 mg/kg 5 mg	Prolonged PFS and OS versus sunitinib which was in association with below-median NLR	[199, 229]
Atezolizumab A2AR antagonist	1	68	840 mg 50–100 mg	A durable clinical benefit associated with increased CTLs infiltration into the tumor	[230]

Note: Programmed cell death ligand 1 (PD-L1), Renal cell carcinoma (RCC), Overall survival (OS), Objective response rate (ORR), Progression-free survival (PFS), Adenosine A2A receptor (A2AR), Treatment-related adverse events (TRAEs), Neutrophil–lymphocyte ratio (NLR), Cytotoxic T cells (CTLs)

## Small molecule compounds inhibiting PD-1/PD-L1 interactions

The restricted success and shortcoming of antibodies have persuaded investigators to examine more efficient approaches for the negative regulation of the PD-1/PD-L1 axis and expand the capacity of cancer immunotherapy. In light of this, substantial efforts are being made to develop low-molecular-weight agents targeting PD-1/PD-L1 interaction [203]. Currently, several companies, including Bristol Myers Squibb (BMS), Arising International Inc, Guangzhou Maxinovel Pharmaceuticals Co, Chemocentryx Inc, Institute of Materia Medica, Incyte Corporation, and Aurigene, have industrialized a variety of small-molecule chemical compounds as well as peptides [204]. Such companies have applied for a series of patents related to inhibitors. These patents offered the structure of PD-1/PD-L1 inhibitors, compound synthesis strategies, and their application as immunomodulators [205]. Further, the patents demonstrate the approved inhibitory impacts of these inhibitors. While some of the evolved small molecule compounds might only deter PD-L1/PD-1 interactions, other inhibitors (e.g., peptides invented by BMS Company) suppress PD-L1 interactions with PD-1 or B7-1 [206]. All inhibitors advanced by Aurigene, such as small molecule chemical compounds and peptides, demonstrated significant inhibitory impact on the PD-1 signaling axis [207]. Notably, most of them demonstrated IC50 values of 1 μM or even 0.018 μM as determined by the PD-1/PD-L1 homogenous time-resolved fluorescence (HTRF) binding assay [204]. Of course, the progress of small molecule compounds inhibiting PD-1/PD-L1 interactions has only just been ongoing. Most of these inhibitors are studied in preclinical studies and are associated with stimulating outcomes [208]. Meanwhile, CA-170, a PD-L1 inhibitor developed by Aurigene and Curis, has arrived phase I clinical trial [209]. Further focus on these novel types of PD-/PD-L1 inhibitors may result in groundbreaking progress in the next future.

## Conclusion and future direction

The treatment setting of advanced RCC has progressed in the last years with emerging ICIs accompanied by the advancement development of novel anti-angiogenic drugs and other therapeutics (Tables 3 and 4). This progress brought about the amelioration of prognosis and improvement of OS and PFS in advanced RCC patients. Nevertheless, there is no head-to-head trial proof to compare the efficacy of the several therapeutic modalities available comprising ICIs, TKIs, or a combination of both. Facts from the further prospective investigation are requisite to directly compare the clinical advantage of ICI in the treatment of clear cell RCC and various subtypes of non-clear cell RCC.

**Table 3:** Completed clinical trials based on monotherapy with anti-PD-1/PD-L1 therapy for renal cell carcinoma (RCC) registered in ClinicalTrials.gov (June 2022)

Agents	Phase	Participant number	Allocation	Dose	Location	NCT number
Nivolumab	2	730	N/A	3 mg/kg	France	NCT03013335
Nivolumab	1	17	N/A	3 mg/kg	USA	NCT02575222
Nivolumab	4	197	N/A	3 mg/kg	USA	NCT02596035
Nivolumab	3	1068	Randomized	3 mg/kg	International	NCT01668784
Nivolumab	2	68	Randomized	0.3–10 mg/kg	International	NCT01354431
Nivolumab	1	395	Non-Randomized	0.1–10 mg/kg	USA	NCT00730639
Nivolumab	1	39	Non-Randomized	0.3–10 mg/kg	USA	NCT00441337
Pembrolizumab	2	275	Non-Randomized	200 mg	UK	NCT02853344

NA

**Table 4:** Completed clinical trials based on combination therapy with anti-PD-1/PD-L1 therapy for renal cell carcinoma (RCC) registered in ClinicalTrials.gov (June 2022)

Agents	Phase	Participant number	Allocation	Dose	Location	NCT number
Nivolumab Ipilimumab	2	118	Randomized	–	USA Australia Chile	NCT03029780
Nivolumab Ipilimumab Sunitinib Pazopanib	1	194	Non-Randomized	5.0 mg/kg 1 mg 50 mg 800 mg	Canada USA	NCT01472081
X4P-001 Nivolumab	1/2	9	N/A	400 mg 240 mg	USA	NCT02923531
IL-2 Nivolumab	1/2	13	N/A	600,000 IU/kg 240 mg	USA	NCT02989714
Nivolumab SBRT	2	69	N/A	240 mg 30 Gy	Italy	NCT03469713
Nivolumab Ipilimumab Sunitinib Pazopanib	2	200	Randomized	5.0 mg/kg 1 mg 50 mg 800 mg	France	NCT02960906
Nivolumab Ipilimumab	4	211	Non-Randomized	–	USA	NCT02982954
Nivolumab CB-839	1/2	118	Non-Randomized	–	USA	NCT02771626
Nivolumab Ipilimumab SBRT	2	29	N/A	–	USA	NCT03065179
Ibrutinib Nivolumab	1/2	31	N/A	–	USA	NCT02899078
Tivozanib Nivolumab	1/2	28	N/A	– 240 mg	France	NCT03136627
Varlilumab Nivolumab	1/2	175	N/A	3 mg/kg 240 mg	USA	NCT02335918
Cabiralizumab Nivolumab	1	313	Non-Randomized	2 mg/kg 3 mg/kg	USA	NCT02526017
Nivolumab ABI-009	1/2	34	N/A	3 mg/kg 100 mg/m^2^	USA	NCT03190174
Pembrolizumab Axitinib	1	52	N/A	2 mg/kg 3–5 mg	USA	NCT02133742
Bevacizumab Pembrolizumab	1/2	61	Non-Randomized	10 mg 200 mg	USA	NCT02348008
Pembrolizumab Ipilimumab or PegIFN-2b	1/2	295	Randomized	200 mg 50–100 mg –	UK	NCT02089685
Pazopanib Pembrolizumab	1	42	Randomized	200 mg 10 mg/kg	USA	NCT02014636
Pembrolizumab Radiotherapy	1/2	30	N/A	200 mg 18–20 Gy	Australia	NCT02855203
Pembrolizumab INCB050465 Itacitinib	1	159	Randomized	200 mg – –	USA	NCT02646748
Pembrolizumab INCB024360	1/2	444	Non-Randomized	25 mg –	USA	NCT02178722
Atezolizumab Bevacizumab	3	915	Randomized	1200 mg 15 mg/kg	International	NCT02420821
Atezolizumab Bevacizumab	2	305	Randomized	1200 mg 15 mg/kg	International	NCT01984242
Atezolizumab Bevacizumab RO6874281	1	69	Randomized	840 mg 10 mg/kg 5 mg	International	NCT03063762
Ciforadenant Atezolizumab	1	502	Randomized	100–200 mg –	Canada USA Australia	NCT02655822
Avelumab Cabozantinib	1	12	N/A	10 mg/kg 20–60 mg	USA	NCT03200587
Avelumab Axitinib	1	55	N/A	5–10 mg/kg 3–5 mg	Japan USA UK	NCT02493751
Durvalumab Tremelimumab	1	29	N/A	– –	USA	NCT02762006
MEDI0680 Durvalumab Nivolumab	1/2	97	Randomized	0.1–20 mg/kg 3–10 mg/kg 240 mg	International	NCT02118337
PolyICLC Durvalumab Tremelimumab	1/2	58	Non-Randomized	–	USA	NCT02643303

NA

Several reports have tried to predict ICIs’ response exploiting several parameters, including the clinical features, laboratory parameters (e.g., NLR), lactate dehydrogenase (LDH), tumor markers, and genetic landscape [210]. Most of them have caused a poor performance because of the absence of comprehensive evaluation in risk stratification. Recent investigations have exhibited that the anti-tumor response to ICIs is a multifaceted process complicating several factors. Previous reports have evolved various prognostic models for prognostic evaluation in ICIs therapy. For instance, a risk scoring criteria comprising monocyte-to-lymphocyte ratio (MLR), sites of metastasis, and nutritional index–body mass index (BMI) were progressed for various for human tumors, in particular RCC patients, who received ICIs [211]. The International Metastatic RCC Database Consortium (IMDC) prognostic risk model remains pivotal in directing treatment selection. Consideration of the durability of treatment response also is urgently required because of the lacking long-term follow-up evidence to validate the durable response and survival merits provided by treatment with dual ICIs therapy. A diversity of clinical trials examining several treatment regimens with ICIs and TKI have shorter follow-up and immature long-term information. Thus, it is ambiguous whether this treatment also delivers comparable durable responses once compared to dual ICIs therapy. Toxicity is also a critical point due to the higher rates of untoward toxicities and the necessity for high-dose corticosteroid treatment associated with dual ICIs therapy when compared to treatment with ICI and anti-angiogenic agents. Mapping the cell types and molecules existing in the TME will potentiate the progress of more effective therapeutic approaches and teach us how to combine currently available options. Finally, a better understanding of the mechanism of adjusting dynamic PD-L1 expression is useful for emerging innovative plans to recover the efficacy of anti-PD-1/PD-L1 compounds.

## Data Availability

Not applicable.

## Abbreviations

PD-1: Programmed cell death protein 1
PD-L1: Programmed cell death ligand 1
ICIs: Immune checkpoint inhibitors
RCC: Renal cell carcinoma
TKI: Tyrosine kinase inhibitor
VEGF: Vascular endothelial growth factor
CTLs: Cytotoxic T-lymphocytes
Tregs: Regulatory T cells
FDA: Food and Drug Administration
TME: Tumor microenvironment
OS: Overall survival
ORR: Objective response rate
PFS: Progression-free survival

## References

[1] Hsieh JJ, Purdue MP, Signoretti S, Swanton C, Albiges L, Schmidinger M, Heng DY (2017). Renal cell carcinoma. Nat Rev Dis Primers.

[2] Turco F, Tucci M, Di Stefano RF, Samuelly A, Bungaro M, Audisio M, Pisano C (2021). Renal cell carcinoma (RCC): fatter is better? A review on the role of obesity in RCC. Endocr Relat Cancer.

[3] Fottner A, Szalantzy M, Wirthmann L, Stähler M, Baur-Melnyk A, Jansson V, Dürr HR (2010). Bone metastases from renal cell carcinoma: patient survival after surgical treatment. BMC Musculoskelet Disord.

[4] Jackson RJ, Gokaslan ZL, Arvinloh S-C (2001). Metastatic renal cell carcinoma of the spine: surgical treatment and results. J Neurosurg Spine.

[5] Fogli S, Porta C, Del Re M, Crucitta S, Gianfilippo G, Danesi R, Rini BI (2020). Optimizing treatment of renal cell carcinoma with VEGFR-TKIs: a comparison of clinical pharmacology and drug-drug interactions of anti-angiogenic drugs. Cancer Treat Rev.

[6] Brighi N, Farolfi A, Conteduca V, Gurioli G, Gargiulo S, Gallà V, Schepisi G (2019). The interplay between inflammation, anti-angiogenic agents, and immune checkpoint inhibitors: perspectives for renal cell cancer treatment. Cancers.

[7] Virumbrales-Muñoz M, Ayuso JM, Loken JR, Denecke KM, Rehman S, Skala MC, Abel EJ (2022). Microphysiological model of renal cell carcinoma to inform anti-angiogenic therapy. Biomaterials.

[8] Massari F, Rizzo A, Mollica V, Rosellini M, Marchetti A, Ardizzoni A, Santoni M (2021). Immune-based combinations for the treatment of metastatic renal cell carcinoma: a meta-analysis of randomised clinical trials. Eur J Cancer.

[9] Şenbabaoğlu Y, Gejman RS, Winer AG, Liu M, Van Allen EM, de Velasco G, Miao D (2016). Tumor immune microenvironment characterization in clear cell renal cell carcinoma identifies prognostic and immunotherapeutically relevant messenger RNA signatures. Genome Biol.

[10] Geissler K, Fornara P, Lautenschläger C, Holzhausen HJ, Seliger B, Riemann D (2015). Immune signature of tumor infiltrating immune cells in renal cancer. Oncoimmunology.

[11] Toor SM, Nair VS, Decock J, Elkord E (2020). Immune checkpoints in the tumor microenvironment. Seminars Cancer Biol.

[12] Toor SM, Murshed K, Al-Dhaheri M, Khawar M, Abu Nada M, Elkord E (2019). Immune checkpoints in circulating and tumor-infiltrating CD4+ T cell subsets in colorectal cancer patients. Front Immunol.

[13] Vafaei S, Zekiy AO, Khanamir RA, Zaman BA, Ghayourvahdat A, Azimizonuzi H, Zamani M (2022). Combination therapy with immune checkpoint inhibitors (ICIs); a new frontier. Cancer Cell Int.

[14] Naimi A, Mohammed RN, Raji A, Chupradit S, Yumashev AV, Suksatan W, Shalaby MN (2022). Tumor immunotherapies by immune checkpoint inhibitors (ICIs); the pros and cons. Cell Commun Signal.

[15] Atkins M, Clark J, Quinn D (2017). Immune checkpoint inhibitors in advanced renal cell carcinoma: experience to date and future directions. Ann Oncol.

[16] Albiges L, Powles T, Staehler M, Bensalah K, Giles RH, Hora M, Kuczyk MA (2019). Updated European Association of Urology guidelines on renal cell carcinoma: immune checkpoint inhibition is the new backbone in first-line treatment of metastatic clear-cell renal cell carcinoma. Eur Urol.

[17] Brinkmann O, Bruns F, Prott F, Hertle L (1999). Possible synergy of radiotherapy and chemo-immunotherapy in metastatic renal cell carcinoma (RCC). Anticancer Res.

[18] De Riese W, Goldenberg K, Allhoff E, Stief C, Schlick R, Liedke S, Jonas U (1991). Metastatic renal cell carcinoma (RCC): spontaneous regression, long-term survival and late recurrence. Int Urol Nephrol.

[19] Braun DA, Ishii Y, Walsh AM, Van Allen EM, Wu CJ, Shukla SA, Choueiri TK (2019). Clinical validation of PBRM1 alterations as a marker of immune checkpoint inhibitor response in renal cell carcinoma. JAMA Oncol.

[20] Hargadon KM, Johnson CE, Williams CJ (2018). Immune checkpoint blockade therapy for cancer: an overview of FDA-approved immune checkpoint inhibitors. Int Immunopharmacol.

[21] Incorvaia L, Madonia G, Corsini LR, Cucinella A, Brando C, Gagliardo C, Santoni M (2021). Challenges and advances for the treatment of renal cancer patients with brain metastases: from immunological background to upcoming clinical evidence on immune-checkpoint inhibitors. Crit Rev Oncol Hematol.

[22] Motzer RJ, Escudier B, McDermott DF, George S, Hammers HJ, Srinivas S, Tykodi SS (2015). Nivolumab versus everolimus in advanced renal-cell carcinoma. N Engl J Med.

[23] Kuusk T, Albiges L, Escudier B, Grivas N, Haanen J, Powles T, Bex A (2017). Antiangiogenic therapy combined with immune checkpoint blockade in renal cancer. Angiogenesis.

[24] Hamilton G (2021). Avelumab: search for combinations of immune checkpoint inhibition with chemotherapy. Expert Opin Biol Ther.

[25] Diegmann J, Junker K, Loncarevic IF, Michel S, Schimmel B, von Eagelinq F (2006). Immune escape for renal cell carcinoma: CD70 mediates apoptosis in lymphocytes. Neoplasia.

[26] Atkins D, Ferrone S, Schmahl GE, Störkel S, Seliger B (2004). Down-regulation of HLA class I antigen processing molecules: an immune escape mechanism of renal cell carcinoma?. J Urol.

[27] Fu Q, Xu L, Wang Y, Jiang Q, Liu Z, Zhang J, Zhou Q (2019). Tumor-associated macrophage-derived interleukin-23 interlinks kidney cancer glutamine addiction with immune evasion. Eur Urol.

[28] Kwaśniak K, Czarnik-Kwaśniak J, Maziarz A, Aebisher D, Zielińska K, Karczmarek-Borowska B, Tabarkiewicz J (2019). Scientific reports concerning the impact of interleukin 4, interleukin 10 and transforming growth factor β on cancer cells. Central-Eur J Immunol.

[29] Salazar-Onfray F, López MN, Mendoza-Naranjo A (2007). Paradoxical effects of cytokines in tumor immune surveillance and tumor immune escape. Cytokine Growth Factor Rev.

[30] Dong P, Xiong Y, Yue J, Hanley SJ, Watari H (2018). Tumor-intrinsic PD-L1 signaling in cancer initiation, development and treatment: beyond immune evasion. Front Oncol.

[31] Ryan AE, Shanahan F, O'Connell J, Houston AM (2005). Addressing the “Fas counterattack” controversy: blocking fas ligand expression suppresses tumor immune evasion of colon cancer in vivo. Can Res.

[32] Liu Y, Cao X (2015). The origin and function of tumor-associated macrophages. Cell Mol Immunol.

[33] Chen DS, Mellman I (2013). Oncology meets immunology: the cancer-immunity cycle. Immunity.

[34] Nagorsen D, Scheibenbogen C, Marincola FM, Letsch A, Keilholz U (2003). Natural T cell immunity against cancer. Clin Cancer Res.

[35] Liu X, Hogg GD, DeNardo DG (2021). Rethinking immune checkpoint blockade: ‘Beyond the T cell’. J Immuno Ther Cancer..

[36] Tsai H-F, Hsu P-N (2017). Cancer immunotherapy by targeting immune checkpoints: mechanism of T cell dysfunction in cancer immunity and new therapeutic targets. J Biomed Sci.

[37] Darvin P, Toor SM, Sasidharan Nair V, Elkord E (2018). Immune checkpoint inhibitors: recent progress and potential biomarkers. Exp Mol Med.

[38] Kong X. Discovery of new immune checkpoints: family grows up. Regulation of Cancer Immune Checkpoints. 2020:61–82. 10.1007/978-981-15-3266-5_4 32185707

[39] Bour-Jordan H, Bluestone JA (2009). Regulating the regulators: costimulatory signals control the homeostasis and function of regulatory T cells. Immunol Rev.

[40] Liu Y, Chen P, Wang H, Wu S, Zhao S, He Y, Zhou C (2021). The landscape of immune checkpoints expression in non-small cell lung cancer: a narrative review. Transl Lung Cancer Res.

[41] Zhang T, Austin RG, Park SE, Runyambo D, Boominathan R, Rao C, Bronson E, et al. Expression of immune checkpoints (ICs) on circulating tumor cells (CTCs) in men with metastatic prostate cancer (mPC). American Society of Clinical Oncology; 2018.

[42] Filippone A, Lanza M, Mannino D, Raciti G, Colarossi C, Sciacca D, Cuzzocrea S (2022). PD1/PD-L1 immune checkpoint as a potential target for preventing brain tumor progression. Cancer Immunol Immunother.

[43] Lee DY, Im E, Yoon D, Lee Y-S, Kim G-S, Kim D, Kim S-H, editors. Pivotal role of PD-1/PD-L1 immune checkpoints in immune escape and cancer progression: Their interplay with platelets and FOXP3+ Tregs related molecules, clinical implications and combinational potential with phytochemicals. Seminars in Cancer Biology; 2020: Elsevier. 10.1016/j.semcancer.2020.12.001 33301862

[44] Gao X, McDermott DF (2018). Ipilimumab in combination with nivolumab for the treatment of renal cell carcinoma. Expert Opin Biol Ther.

[45] Motzer RJ, Tannir NM, McDermott DF, Frontera OA, Melichar B, Choueiri TK, Plimack ER (2018). Nivolumab plus ipilimumab versus sunitinib in advanced renal-cell carcinoma. N Engl J Med.

[46] Chulpanova DS, Kitaeva KV, Green AR, Rizvanov AA, Solovyeva VV (2020). Molecular aspects and future perspectives of cytokine-based anti-cancer immunotherapy. Front Cell Dev Biol.

[47] Berraondo P, Sanmamed MF, Ochoa MC, Etxeberria I, Aznar MA, Pérez-Gracia JL, Rodríguez-Ruiz ME (2019). Cytokines in clinical cancer immunotherapy. Br J Cancer.

[48] West W (1989). Continuous infusion recombinant interleukin-2 (rIL-2) in adoptive cellular therapy of renal carcinoma and other malignancies. Cancer Treat Rev.

[49] Spolski R, Li P, Leonard WJ (2018). Biology and regulation of IL-2: from molecular mechanisms to human therapy. Nat Rev Immunol.

[50] Rosenberg SA (2014). IL-2: the first effective immunotherapy for human cancer. J Immunol.

[51] Mortara L, Balza E, Bruno A, Poggi A, Orecchia P, Carnemolla B (2018). Anti-cancer therapies employing IL-2 cytokine tumor targeting: contribution of innate, adaptive and immunosuppressive cells in the anti-tumor efficacy. Front Immunol.

[52] Alva A, Daniels GA, Wong MKK, Kaufman HL, Morse MA, McDermott DF, Clark JI (2016). Contemporary experience with high-dose interleukin-2 therapy and impact on survival in patients with metastatic melanoma and metastatic renal cell carcinoma. Cancer Immunol Immunother.

[53] Fyfe G, Fisher RI, Rosenberg SA, Sznol M, Parkinson DR, Louie AC (1995). Results of treatment of 255 patients with metastatic renal cell carcinoma who received high-dose recombinant interleukin-2 therapy. J Clin Oncol.

[54] Achkar T, Arjunan A, Wang H, Saul M, Davar D, Appleman LJ, Friedland D (2017). High-dose interleukin 2 in patients with metastatic renal cell carcinoma with sarcomatoid features. PLoS ONE.

[55] Huland E, Heinzer H, Huland H, Yung R (2000). Overview of interleukin-2 inhalation therapy. Cancer J Sci Am.

[56] Choudhry H, Helmi N, Abdulaal WH, Zeyadi M, Zamzami MA, Wu W, Mahmoud MM (2018). Prospects of IL-2 in cancer immunotherapy. BioMed Res Int.

[57] Cerbone L, Cattrini C, Vallome G, Latocca MM, Boccardo F, Zanardi E (2020). Combination therapy in metastatic renal cell carcinoma: back to the future?. Semin Oncol.

[58] Passalacqua R, Caminiti C, Buti S, Porta C, Camisa R, Braglia L, Tomasello G (2014). Adjuvant low-dose interleukin-2 (IL-2) plus interferon-α (IFN-α) in operable renal cell carcinoma (RCC): a phase III, randomized, multicentre trial of the Italian Oncology Group for Clinical Research (GOIRC). J Immunother.

[59] Westermann J, Reich G, Kopp J, Haus U, Dörken B, Pezzutto A (2001). Granulocyte/macrophage-colony-stimulating-factor plus interleukin-2 plus interferon alpha in the treatment of metastatic renal cell carcinoma: a pilot study. Cancer Immunol Immunother.

[60] Smith IJ, Kurt RA, Baher AG, Denman S, Justice L, Doran T, Gilbert M (2003). Immune effects of escalating doses of granulocyte-macrophage colony-stimulating factor added to a fixed, low-dose, inpatient interleukin-2 regimen: a randomized phase I trial in patients with metastatic melanoma and renal cell carcinoma. J Immunother.

[61] Hannan R, Mohamad O, Diaz de Leon A, Manna S, Pop LM, Zhang Z, Mannala S (2021). Outcome and immune correlates of a Phase II trial of high-dose interleukin-2 and stereotactic ablative radiotherapy for metastatic renal cell carcinoma. Clin Cancer Res.

[62] Göhring B, Riemann D, Rebmann U, Heynemann H, Schabel J, Langner J (1996). Prognostic value of the immunomonitoring of patients with renal cell carcinoma under therapy with IL-2/IFN-alpha-2 in combination with 5-FU. Urol Res.

[63] Vergati M, Intrivici C, Huen N-Y, Schlom J, Tsang KY (2010). Strategies for cancer vaccine development. J Biomed Biotechnol.

[64] Zhang Y, Ma S, Liu X, Xu Y, Zhao J, Si X, Li H (2021). Supramolecular assembled programmable nanomedicine as in situ cancer vaccine for cancer immunotherapy. Adv Mater.

[65] Qin H, Zhao R, Qin Y, Zhu J, Chen L, Di C, Han X (2021). Development of a cancer vaccine using in vivo click-chemistry-mediated active lymph node accumulation for improved immunotherapy. Adv Mater.

[66] Tanyi JL, Chiang CL-L, Chiffelle J, Thierry A-C, Baumgartener P, Huber F, Goepfert C (2021). Personalized cancer vaccine strategy elicits polyfunctional T cells and demonstrates clinical benefits in ovarian cancer. NPJ Caccines..

[67] Wang T, Wang D, Yu H, Feng B, Zhou F, Zhang H, Zhou L (2018). A cancer vaccine-mediated postoperative immunotherapy for recurrent and metastatic tumors. Nat Commun.

[68] Goldman B, DeFrancesco L (2009). The cancer vaccine roller coaster. Nat Biotechnol.

[69] Hammerstrom AE, Cauley DH, Atkinson BJ, Sharma P (2011). Cancer immunotherapy: sipuleucel-T and beyond. Pharmacotherapy.

[70] Pal SK, Hu A, Figlin RA (2013). A new age for vaccine therapy in renal cell carcinoma. Cancer J.

[71] Jian Y, Yang K, Sun X, Zhao J, Huang K, Aldanakh A, Xu Z (2021). Current advance of immune evasion mechanisms and emerging immunotherapies in renal cell carcinoma. Front Immunol.

[72] Amin A, Dudek AZ, Logan TF, Lance RS, Holzbeierlein JM, Knox JJ, Master VA (2015). Survival with AGS-003, an autologous dendritic cell–based immunotherapy, in combination with sunitinib in unfavorable risk patients with advanced renal cell carcinoma (RCC): phase 2 study results. J Immunother Cancer.

[73] Figlin R, Nicolette C, Tannir N, Tykodi S, Chen D, Master V, Lane B (2017). Interim analysis of the phase 3 ADAPT trial evaluating rocapuldencel-T (AGS-003), an individualized immunotherapy for the treatment of newly-diagnosed patients with metastatic renal cell carcinoma (mRCC). Ann Oncol.

[74] Figlin R, Sternberg C, Wood CG (2012). Novel agents and approaches for advanced renal cell carcinoma. J Urol.

[75] Kirner A, Mayer-Mokler A, Reinhardt C (2014). IMA901: a multi-peptide cancer vaccine for treatment of renal cell cancer. Hum Vaccin Immunother.

[76] Rausch S, Kruck S, Stenzl A, Bedke J (2014). IMA901 for metastatic renal cell carcinoma in the context of new approaches to immunotherapy. Future Oncol.

[77] Rini BI, Stenzl A, Zdrojowy R, Kogan M, Shkolnik M, Oudard S, Weikert S (2016). IMA901, a multipeptide cancer vaccine, plus sunitinib versus sunitinib alone, as first-line therapy for advanced or metastatic renal cell carcinoma (IMPRINT): a multicentre, open-label, randomised, controlled, phase 3 trial. Lancet Oncol.

[78] Birkhäuser FD, Koya RC, Neufeld C, Rampersaud EN, Lu X, Micewicz ED, Chodon T (2013). Dendritic cell-based immunotherapy in prevention and treatment of renal cell carcinoma: efficacy, safety, and activity of Ad-GM·CAIX in immunocompetent mouse models. J Immunother.

[79] Faiena I, Comin-Anduix B, Berent-Maoz B, Bot A, Zomorodian N, Sachdeva A, Said J (2020). A Phase I, open-label, dose-escalation, and cohort expansion study to evaluate the safety and immune response to autologous dendritic cells transduced with AdGMCA9 (DC-AdGMCAIX) in patients with metastatic renal cell carcinoma. J Immunother.

[80] Baek S, Kim CS, Kim SB, Kim YM, Kwon SW, Kim Y, Kim H (2011). Combination therapy of renal cell carcinoma or breast cancer patients with dendritic cell vaccine and IL-2: results from a phase I/II trial. J Transl Med.

[81] Amato RJ, Shetty A, Lu Y, Ellis PR, Mohlere V, Carnahan N, Low PS (2014). A Phase I/Ib study of folate immune (EC90 vaccine administered with GPI-0100 adjuvant followed by EC17) with interferon-α and interleukin-2 in patients with renal cell carcinoma. J Immunother.

[82] Capitini CM, Fry TJ, Mackall CL (2009). Cytokines as adjuvants for vaccine and cellular therapies for cancer. Am J Immunol.

[83] Galluzzi L, Vacchelli E, Eggermont A, Fridman WH, Galon J, Sautès-Fridman C, Tartour E (2012). Trial watch: adoptive cell transfer immunotherapy. Oncoimmunology.

[84] Ma C, Cheung AF, Chodon T, Koya RC, Wu Z, Ng C, Avramis E (2013). Multifunctional T-cell analyses to study response and progression in adoptive cell transfer immunotherapy. Cancer Discov.

[85] Roncati L, Palmieri B (2020). Adoptive cell transfer (ACT) of autologous tumor-infiltrating lymphocytes (TILs) to treat malignant melanoma: the dawn of a chimeric antigen receptor T (CAR-T) cell therapy from autologous donor. Int J Dermatol.

[86] Daher M, Rezvani K (2021). Outlook for new CAR-based therapies with a focus on CAR NK cells: what lies beyond CAR-engineered T cells in the race against cancer. Cancer Discov.

[87] Bachiller M, Perez-Amill L, Battram AM, Carné SC, Najjar A, Verhoeyen E, Juan M (2021). NK cells enhance CAR-T cell antitumor efficacy by enhancing immune/tumor cells cluster formation and improving CAR-T cell fitness. J Immunother Cancer.

[88] Nguyen LT, Yen PH, Nie J, Liadis N, Ghazarian D, Al-Habeeb A, Easson A (2010). Expansion and characterization of human melanoma tumor-infiltrating lymphocytes (TILs). PLoS ONE.

[89] Rosenberg SA, Yang JC, Sherry RM, Kammula US, Hughes MS, Phan GQ, Citrin DE (2011). Durable complete responses in heavily pretreated patients with metastatic melanoma using T-cell transfer immunotherapy. Clin Cancer Res.

[90] Lorentzen C, Straten P (2015). CD 19-chimeric antigen receptor T cells for treatment of chronic lymphocytic leukaemia and acute lymphoblastic leukaemia. Scand J Immunol.

[91] Bear AS, Fraietta JA, Narayan VK, O’Hara M, Haas NB (2021). Adoptive cellular therapy for solid tumors. Am Soc Clin Oncol Educ Book.

[92] Andersen R, Westergaard MCW, Kjeldsen JW, Müller A, Pedersen NW, Hadrup SR, Met Ö (2018). T-cell responses in the microenvironment of primary renal cell carcinoma—implications for adoptive cell therapy. Cancer Immunol Res.

[93] Figlin RA, Thompson JA, Bukowski RM, Vogelzang NJ, Novick AC, Lange P, Steinberg GD (1999). Multicenter, randomized, phase III trial of CD8+ tumor-infiltrating lymphocytes in combination with recombinant interleukin-2 in metastatic renal cell carcinoma. J Clin Oncol.

[94] Lamers CH, Klaver Y, Gratama JW, Sleijfer S, Debets R (2016). Treatment of metastatic renal cell carcinoma (mRCC) with CAIX CAR-engineered T-cells–a completed study overview. Biochem Soc Trans.

[95] Lamers CH, Sleijfer S, Van Steenbergen S, Van Elzakker P, Van Krimpen B, Groot C, Vulto A (2013). Treatment of metastatic renal cell carcinoma with CAIX CAR-engineered T cells: clinical evaluation and management of on-target toxicity. Mol Ther.

[96] Panowski SH, Srinivasan S, Tan N, Tacheva-Grigorova SK, Smith B, Mak YS, Ning H, et al. Preclinical development and evaluation of allogeneic CAR T cells targeting CD70 for the treatment of renal cell carcinoma. Cancer Res. 2022:OF1-OF15. 10.1158/0008-5472.CAN-21-2931 35294525

[97] Mori J, Adachi K, Sakoda Y, Sasaki T, Goto S, Matsumoto H, Nagashima Y (2021). Anti-tumor efficacy of human anti-c-met CAR-T cells against papillary renal cell carcinoma in an orthotopic model. Cancer Sci.

[98] Escudier B (2012). Emerging immunotherapies for renal cell carcinoma. Ann Oncol.

[99] Moreira M, Pobel C, Epaillard N, Simonaggio A, Oudard S, Vano Y-A (2020). Resistance to cancer immunotherapy in metastatic renal cell carcinoma. Cancer Drug Resist.

[100] Bai D, Feng H, Yang J, Yin A, Qian A, Sugiyama H (2021). Landscape of immune cell infiltration in clear cell renal cell carcinoma to aid immunotherapy. Cancer Sci.

[101] Ishida Y, Agata Y, Shibahara K, Honjo T (1992). Induced expression of PD-1, a novel member of the immunoglobulin gene superfamily, upon programmed cell death. Embo j.

[102] Nishimura H, Nose M, Hiai H, Minato N, Honjo T (1999). Development of lupus-like autoimmune diseases by disruption of the PD-1 gene encoding an ITIM motif-carrying immunoreceptor. Immunity.

[103] Fan P, Li X, Feng Y, Cai H, Dong D, Peng Y, Yao X (2021). PD-1 expression status on CD8+ tumour infiltrating lymphocytes associates with survival in cervical cancer. Front Oncol.

[104] Davis Z, Felices M, Lenvik T, Badal S, Walker JT, Hinderlie P, Riley JL (2021). Low-density PD-1 expression on resting human natural killer cells is functional and upregulated after transplantation. Blood Adv.

[105] Judge SJ, Dunai C, Aguilar EG, Vick SC, Sturgill IR, Khuat LT, Stoffel KM (2020). Minimal PD-1 expression in mouse and human NK cells under diverse conditions. J Clin Investig.

[106] Lim TS, Chew V, Sieow JL, Goh S, Yeong JP-S, Soon AL, Ricciardi-Castagnoli P (2016). PD-1 expression on dendritic cells suppresses CD8+ T cell function and antitumor immunity. Oncoimmunology..

[107] Kuipers H, Muskens F, Willart M, Hijdra D, van Assema FB, Coyle AJ, Hoogsteden HC (2006). Contribution of the PD-1 ligands/PD-1 signaling pathway to dendritic cell-mediated CD4+ T cell activation. Eur J Immunol.

[108] Han Y, Liu D, Li L (2020). PD-1/PD-L1 pathway: current researches in cancer. Am J Cancer Res.

[109] Patsoukis N, Duke-Cohan JS, Chaudhri A, Aksoylar H-I, Wang Q, Council A, Berg A (2020). Interaction of SHP-2 SH2 domains with PD-1 ITSM induces PD-1 dimerization and SHP-2 activation. Commun Biol.

[110] Veluswamy P, Wacker M, Scherner M, Wippermann J (2020). Delicate role of PD-L1/PD-1 axis in blood vessel inflammatory diseases: current insight and future significance. Int J Mol Sci.

[111] Cretella D, Digiacomo G, Giovannetti E, Cavazzoni A (2019). PTEN alterations as a potential mechanism for tumor cell escape from PD-1/PD-L1 inhibition. Cancers.

[112] Zitvogel L, Kroemer G (2012). Targeting PD-1/PD-L1 interactions for cancer immunotherapy. Oncoimmunology.

[113] Good-Jacobson KL, Szumilas CG, Chen L, Sharpe AH, Tomayko MM, Shlomchik MJ (2010). PD-1 regulates germinal center B cell survival and the formation and affinity of long-lived plasma cells. Nat Immunol.

[114] Hudson K, Cross N, Jordan-Mahy N, Leyland R (2020). The extrinsic and intrinsic roles of PD-L1 and its receptor PD-1: implications for immunotherapy treatment. Front Immunol.

[115] Chiu Y-M, Tsai C-L, Kao J-T, Hsieh C-T, Shieh D-C, Lee Y-J, Tsay GJ (2018). PD-1 and PD-L1 up-regulation promotes T-cell apoptosis in gastric adenocarcinoma. Anticancer Res.

[116] Zheng H, Ning Y, Zhan Y, Liu S, Wen Q, Fan S (2021). New insights into the important roles of tumor cell-intrinsic PD-1. Int J Biol Sci.

[117] Iacovelli R, Nolè F, Verri E, Renne G, Paglino C, Santoni M, Cossu Rocca M (2016). Prognostic role of PD-L1 expression in renal cell carcinoma. A systematic review and meta-analysis. Target Oncol.

[118] Shen M, Chen G, Xie Q, Li X, Xu H, Wang H, Zhao S (2020). Association between PD-L1 expression and the prognosis and clinicopathologic features of renal cell carcinoma: a systematic review and meta-analysis. Urol Int.

[119] Kumar A, Chamoto K (2021). Immune metabolism in PD-1 blockade-based cancer immunotherapy. Int Immunol.

[120] Sun L, Zhang L, Yu J, Zhang Y, Pang X, Ma C, Shen M (2020). Clinical efficacy and safety of anti-PD-1/PD-L1 inhibitors for the treatment of advanced or metastatic cancer: a systematic review and meta-analysis. Sci Rep.

[121] Ancevski Hunter K, Socinski MA, Villaruz LC (2018). PD-L1 testing in guiding patient selection for PD-1/PD-L1 inhibitor therapy in lung cancer. Mol Diagn Ther.

[122] Gunturi A, McDermott DF (2015). Nivolumab for the treatment of cancer. Expert Opin Investig Drugs.

[123] Mazza C, Escudier B, Albiges L (2017). Nivolumab in renal cell carcinoma: latest evidence and clinical potential. Ther Adv Med Oncol.

[124] Choueiri TK, Powles T, Burotto M, Escudier B, Bourlon MT, Zurawski B, Oyervides Juárez VM (2021). Nivolumab plus cabozantinib versus sunitinib for advanced renal-cell carcinoma. N Engl J Med.

[125] Tannir NM, Signoretti S, Choueiri TK, McDermott DF, Motzer RJ, Flaifel A, Pignon J-C (2021). Efficacy and safety of nivolumab plus ipilimumab versus sunitinib in first-line treatment of patients with advanced sarcomatoid renal cell carcinoma. Clin Cancer Res.

[126] Weight C. Nivolumab versus everolimus in advanced renal cell carcinoma. 50 Studies Every Urologist Should Know. 2021:123.

[127] Escudier B, Sharma P, McDermott DF, George S, Hammers HJ, Srinivas S, Tykodi SS (2017). CheckMate 025 randomized phase 3 study: outcomes by key baseline factors and prior therapy for nivolumab versus everolimus in advanced renal cell carcinoma. Eur Urol.

[128] Escudier B, Motzer RJ, Sharma P, Wagstaff J, Plimack ER, Hammers HJ, Donskov F (2017). Treatment beyond progression in patients with advanced renal cell carcinoma treated with nivolumab in CheckMate 025. Eur Urol.

[129] Motzer RJ, Escudier B, George S, Hammers HJ, Srinivas S, Tykodi SS, Sosman JA (2020). Nivolumab versus everolimus in patients with advanced renal cell carcinoma: updated results with long-term follow-up of the randomized, open-label, phase 3 CheckMate 025 trial. Cancer.

[130] Mollica V, Rizzo A, Tassinari E, Giunchi F, Schiavina R, Fiorentino M, Brunocilla E (2021). Prognostic and predictive factors to nivolumab in patients with metastatic renal cell carcinoma: a single center study. Anticancer Drugs.

[131] McFarlane JJ, Kochenderfer MD, Olsen MR, Bauer TM, Molina A, Hauke RJ, Reeves JA (2020). Safety and efficacy of nivolumab in patients with advanced clear cell renal cell carcinoma: results from the phase IIIb/IV CheckMate 374 study. Clin Genitourinary Cancer..

[132] Suzuki K, Terakawa T, Furukawa J, Harada K, Hinata N, Nakano Y, Fujisawa M (2020). Clinical outcomes of second-line treatment following prior targeted therapy in patients with metastatic renal cell carcinoma: a comparison of axitinib and nivolumab. Int J Clin Oncol.

[133] Albiges L, Tannir NM, Burotto M, McDermott D, Plimack ER, Barthélémy P, Porta C (2020). Nivolumab plus ipilimumab versus sunitinib for first-line treatment of advanced renal cell carcinoma: extended 4-year follow-up of the phase III CheckMate 214 trial. ESMO open.

[134] Hammers HJ, Plimack ER, Infante JR, Rini BI, McDermott DF, Lewis LD, Voss MH (2017). Safety and efficacy of nivolumab in combination with ipilimumab in metastatic renal cell carcinoma: the CheckMate 016 study. J Clin Oncol.

[135] Choueiri TK, Apolo AB, Powles T, Escudier B, Aren OR, Shah A, Kessler ER, et al. A phase 3, randomized, open-label study of nivolumab combined with cabozantinib vs sunitinib in patients with previously untreated advanced or metastatic renal cell carcinoma (RCC; CheckMate 9ER). Am Soc Clin Oncol. 2018.

[136] Kfoury M, Oing C (2021). ESMO20 YO for YO: highlights on metastatic renal cell carcinoma—the CheckMate-9ER trial. ESMO Open..

[137] Khalil N, Sarkis J, Abi Tayeh G. Use of immunotherapy with programmed cell death 1 vs programmed cell death ligand 1 in renal cell carcinoma: lessons from CheckMate 9ER and IMmotion 151. SAGE Publications Sage UK: London, England; 2021. p. 266–7. 10.1177/1078155221989424 33470172

[138] Amin A, Plimack ER, Ernstoff MS, Lewis LD, Bauer TM, McDermott DF, Carducci M (2018). Safety and efficacy of nivolumab in combination with sunitinib or pazopanib in advanced or metastatic renal cell carcinoma: the CheckMate 016 study. J Immunother Cancer.

[139] Choueiri TK, Atkins MB, Rose TL, Alter RS, Ju Y, Niland K, Wang Y (2021). A phase 1b trial of the CXCR4 inhibitor mavorixafor and nivolumab in advanced renal cell carcinoma patients with no prior response to nivolumab monotherapy. Invest New Drugs.

[140] Diab A, Tannir NM, Bentebibel S-E, Hwu P, Papadimitrakopoulou V, Haymaker C, Kluger HM (2020). Bempegaldesleukin (NKTR-214) plus nivolumab in patients with advanced solid tumors: phase I dose-escalation study of safety, efficacy, and immune activation (PIVOT-02). Cancer Discov.

[141] Dizman N, Meza L, Bergerot P, Alcantara M, Dorff T, Lyou Y, Frankel P (2022). Nivolumab plus ipilimumab with or without live bacterial supplementation in metastatic renal cell carcinoma: a randomized phase 1 trial. Nat Med.

[142] Meza LA, Dizman N, Bergerot PG, Dorff TB, Lyou Y, Frankel PH, Mira V (2021). First results of a randomized phase IB study comparing nivolumab/ipilimumab with or without CBM-588 in patients with metastatic renal cell carcinoma. J Clin Oncol.

[143] Sharma M, Khong H, Fa’ak F, Bentebibel S-E, Janssen LM, Chesson BC, Creasy CA (2020). Bempegaldesleukin selectively depletes intratumoral Tregs and potentiates T cell-mediated cancer therapy. Nat Commun.

[144] Khoja L, Butler MO, Kang SP, Ebbinghaus S, Joshua AM (2015). Pembrolizumab. J Immunother Cancer.

[145] Rini BI, Plimack ER, Stus V, Gafanov R, Hawkins R, Nosov D, Pouliot F (2019). Pembrolizumab plus axitinib versus sunitinib for advanced renal-cell carcinoma. N Engl J Med.

[146] Motzer R, Alekseev B, Rha S-Y, Porta C, Eto M, Powles T, Grünwald V (2021). Lenvatinib plus pembrolizumab or everolimus for advanced renal cell carcinoma. N Engl J Med.

[147] Choueiri TK, Quinn DI, Zhang T, Gurney H, Doshi GK, Cobb PW, Parnis F, et al. KEYNOTE-564: A phase 3, randomized, double blind, trial of pembrolizumab in the adjuvant treatment of renal cell carcinoma. American Society of Clinical Oncology; 2018.

[148] Choueiri TK, Tomczak P, Park SH, Venugopal B, Ferguson T, Chang Y-H, Hajek J, et al. Pembrolizumab versus placebo as post-nephrectomy adjuvant therapy for patients with renal cell carcinoma: Randomized, double-blind, phase III KEYNOTE-564 study. Am Soc Clin Oncol. 2021.

[149] Quinn DI, Zhang T, Gurney H, Doshi GK, Cobb PW, Parnis F, Lee J-L, et al. Phase 3, randomized, double-blind trial of pembrolizumab in the adjuvant treatment of renal cell carcinoma (RCC): KEYNOTE-564. Am Soc Clin Oncol; 2018.

[150] Choueiri TK, Tomczak P, Park SH, Venugopal B, Ferguson T, Chang Y-H, Hajek J (2021). Adjuvant pembrolizumab after nephrectomy in renal-cell carcinoma. N Engl J Med.

[151] McDermott DF, Lee JL, Bjarnason GA, Larkin JM, Gafanov RA, Kochenderfer MD, Jensen NV (2021). Open-label, single-arm phase II study of pembrolizumab monotherapy as first-line therapy in patients with advanced clear cell renal cell carcinoma. J Clin Oncol.

[152] McDermott DF, Lee J-L, Ziobro M, Suárez Rodríguez C, Langiewicz P, Matveev VB (2021). Open-label, single-arm, phase II study of pembrolizumab monotherapy as first-line therapy in patients with advanced non–clear cell renal cell carcinoma. Am Soc Clin Oncol.

[153] Grünwald V, Powles T, Choueiri TK, Hutson TE, Porta C, Eto M, Sternberg CN (2019). Lenvatinib plus everolimus or pembrolizumab versus sunitinib in advanced renal cell carcinoma: study design and rationale. Future Oncol.

[154] Chau V, Bilusic M (2020). Pembrolizumab in combination with axitinib as first-line treatment for patients with renal cell carcinoma (RCC): evidence to date. Cancer Manag Res.

[155] Powles T, Plimack ER, Soulières D, Waddell T, Stus V, Gafanov R, Nosov D (2020). Pembrolizumab plus axitinib versus sunitinib monotherapy as first-line treatment of advanced renal cell carcinoma (KEYNOTE-426): extended follow-up from a randomised, open-label, phase 3 trial. Lancet Oncol.

[156] Atkins MB, Plimack ER, Puzanov I, Fishman MN, McDermott DF, Cho DC, Vaishampayan U (2018). Axitinib in combination with pembrolizumab in patients with advanced renal cell cancer: a non-randomised, open-label, dose-finding, and dose-expansion phase 1b trial. Lancet Oncol.

[157] Zhu J, Zhang T, Wan N, Liang Z, Li J, Chen X, Liang W (2020). Cost–effectiveness of pembrolizumab plus axitinib as first-line therapy for advanced renal cell carcinoma. Immunotherapy.

[158] Colombo N, Lorusso D, Herráez AC, Santin A, Colomba E, Miller D, Fujiwara K (2021). 726MO Outcomes by histology and prior therapy with lenvatinib plus pembrolizumab vs treatment of physician’s choice in patients with advanced endometrial cancer (Study 309/KEYNOTE-775). Ann Oncol.

[159] Chowdhury S, Infante JR, Hawkins R, Voss MH, Perini R, Arkenau T, Voskoboynik M (2021). A Phase I/II Study to Assess the safety and efficacy of pazopanib and pembrolizumab combination therapy in patients with advanced renal cell carcinoma. Clin Genitourin Cancer.

[160] Naing A, Wong DJ, Infante JR, Korn WM, Aljumaily R, Papadopoulos KP, Autio KA (2019). Pegilodecakin combined with pembrolizumab or nivolumab for patients with advanced solid tumours (IVY): a multicentre, multicohort, open-label, phase 1b trial. Lancet Oncol.

[161] Atkins MB, Hodi FS, Thompson JA, McDermott DF, Hwu WJ, Lawrence DP, Dawson NA (2018). Pembrolizumab plus pegylated interferon alfa-2b or ipilimumab for advanced melanoma or renal cell carcinoma: dose-finding results from the phase Ib KEYNOTE-029 Study. Clin Cancer Res.

[162] Hoy SM (2019). Sintilimab: first global approval. Drugs.

[163] Ansell SM (2019). Sintilimab: another effective immune checkpoint inhibitor in classical Hodgkin lymphoma. Lancet Haematol.

[164] Yan Z, Yao S, Liu Y, Zhang J, Li P, Wang H, Chu J (2020). Durable response to sintilimab and chidamide in a patient with pegaspargase-and immunotherapy-resistant NK/T-cell lymphoma: Case report and literature review. Front Oncol.

[165] DU Y. Efficacy of axitinib plus sintilimab in intermediate-and high-risk advanced renal cell carcinoma. Chin J Clin Oncol. 2020:513–6.

[166] Lu X, Gu W, Shi G, Ye D (2021). Pazopanib together with 6–8 cycles of sintilimab followed by single use of pazopanib in the second-line treatment of advanced renal cell carcinoma. Transl Androl Urol.

[167] Méndez-Vidal MJ, Molina Á, Anido U, Chirivella I, Etxaniz O, Fernández-Parra E, Guix M (2018). Pazopanib: evidence review and clinical practice in the management of advanced renal cell carcinoma. BMC Pharmacol Toxicol.

[168] Huang N, Zhao C, Hu X, Zhang C, Xiong F, Huang W, Da L (2022). Safety and efficacy of sintilimab combination therapy for the treatment of 48 patients with advanced malignant tumors. Transl Cancer Res.

[169] Markham A (2016). Atezolizumab: first global approval. Drugs.

[170] Powles T, Durán I, Van der Heijden MS, Loriot Y, Vogelzang NJ, De Giorgi U, Oudard S (2018). Atezolizumab versus chemotherapy in patients with platinum-treated locally advanced or metastatic urothelial carcinoma (IMvigor211): a multicentre, open-label, phase 3 randomised controlled trial. Lancet.

[171] Santini FC, Rudin CM (2017). Atezolizumab for the treatment of non-small cell lung cancer. Expert Rev Clin Pharmacol.

[172] Rittmeyer A, Barlesi F, Waterkamp D, Park K, Ciardiello F, Von Pawel J, Gadgeel SM (2017). Atezolizumab versus docetaxel in patients with previously treated non-small-cell lung cancer (OAK): a phase 3, open-label, multicentre randomised controlled trial. Lancet.

[173] Adams S, Diéras V, Barrios C, Winer E, Schneeweiss A, Iwata H, Loi S (2020). Patient-reported outcomes from the phase III IMpassion130 trial of atezolizumab plus nab-paclitaxel in metastatic triple-negative breast cancer. Ann Oncol.

[174] Kelley RK, Oliver J, Hazra S, Benzaghou F, Yau T, Cheng A-L, Rimassa L (2020). Cabozantinib in combination with atezolizumab versus sorafenib in treatment-naive advanced hepatocellular carcinoma: COSMIC-312 Phase III study design. Future Oncol.

[175] Rini BI, Powles T, Atkins MB, Escudier B, McDermott DF, Suarez C, Bracarda S (2019). Atezolizumab plus bevacizumab versus sunitinib in patients with previously untreated metastatic renal cell carcinoma (IMmotion151): a multicentre, open-label, phase 3, randomised controlled trial. Lancet.

[176] Rini B, Huseni M, Atkins M, McDermott D, Powles T, Escudier B, Banchereau R (2018). Molecular correlates differentiate response to atezolizumab (atezo)+ bevacizumab (bev) vs sunitinib (sun): results from a phase III study (IMmotion151) in untreated metastatic renal cell carcinoma (mRCC). Ann Oncol.

[177] McDermott DF, Sosman JA, Sznol M, Massard C, Gordon MS, Hamid O, Powderly JD (2016). Atezolizumab, an anti-programmed death-ligand 1 antibody, in metastatic renal cell carcinoma: long-term safety, clinical activity, and immune correlates from a phase Ia study. J Clin Oncol.

[178] Uzzo R, Bex A, Rini BI, Albiges L, Suarez C, Donaldson F, Asakawa T, et al. A phase III study of atezolizumab (atezo) vs placebo as adjuvant therapy in renal cell carcinoma (RCC) patients (pts) at high risk of recurrence following resection (IMmotion010). Am Soc Clin Oncol; 2017.

[179] McDermott DF, Huseni MA, Atkins MB, Motzer RJ, Rini BI, Escudier B, Fong L (2018). Clinical activity and molecular correlates of response to atezolizumab alone or in combination with bevacizumab versus sunitinib in renal cell carcinoma. Nat Med.

[180] Pal SK, Albiges L, Suarez Rodriguez C, Liu B, Doss J, Khurana S, Scheffold C, et al. CONTACT-03: Randomized, open-label phase III study of atezolizumab plus cabozantinib versus cabozantinib monotherapy following progression on/after immune checkpoint inhibitor (ICI) treatment in patients with advanced/metastatic renal cell carcinoma. Am Soc Clin Oncol; 2021.

[181] Atkins AB, McDermott DF, Powles T, Motzer RJ, Rini BI, Fong L, Joseph RW (2017). IMmotion150: A phase II trial in untreated metastatic renal cell carcinoma (mRCC) patients (pts) of atezolizumab (atezo) and bevacizumab (bev) vs and following atezo or sunitinib (sun). Am Soc Clin Oncol.

[182] Motzer RJ, Powles T, Atkins MB, Escudier B, McDermott DF, Alekseev BY, Lee J-L (2022). Final overall survival and molecular analysis in immotion151, a phase 3 trial comparing atezolizumab plus bevacizumab vs sunitinib in patients with previously untreated metastatic renal cell carcinoma. JAMA Oncol.

[183] Atkins MB, Rini BI, Motzer RJ, Powles T, McDermott DF, Suarez C, Bracarda S (2020). Patient-reported outcomes from the phase III Randomized IMmotion151 Trial: Atezolizumab+ Bevacizumab versus sunitinib in treatment-naive metastatic renal cell carcinoma. Clin Cancer Res.

[184] Blank CU, Wong DJ, Ho TH, Bauer TM, Lee CB, Bene-Tchaleu F, Zhu J (2021). Phase Ib study of atezolizumab plus interferon-α with or without bevacizumab in patients with metastatic renal cell carcinoma and other solid tumors. Curr Oncol.

[185] Ferrantini M, Capone I, Belardelli F (2007). Interferon-α and cancer: mechanisms of action and new perspectives of clinical use. Biochimie.

[186] Kadowaki N, Antonenko S, Lau JY-N, Liu Y-J (2000). Natural interferon α/β–producing cells link innate and adaptive immunity. J Exp Med.

[187] Rini BI, Halabi S, Rosenberg JE, Stadler WM, Vaena DA, Archer L, Atkins JN (2010). Phase III trial of bevacizumab plus interferon alfa versus interferon alfa monotherapy in patients with metastatic renal cell carcinoma: final results of CALGB 90206. J Clin Oncol.

[188] McDermott DF, George DJ (2010). Bevacizumab as a treatment option in advanced renal cell carcinoma: an analysis and interpretation of clinical trial data. Cancer Treat Rev.

[189] Jung KH, LoRusso P, Burris H, Gordon M, Bang Y-J, Hellmann MD, Cervantes A (2019). Phase I study of the indoleamine 2, 3-dioxygenase 1 (IDO1) inhibitor navoximod (GDC-0919) administered with PD-L1 inhibitor (atezolizumab) in advanced solid tumors. Clin Cancer Res.

[190] Liu M, Wang X, Wang L, Ma X, Gong Z, Zhang S, Li Y (2018). Targeting the IDO1 pathway in cancer: from bench to bedside. J Hematol Oncol.

[191] Zhai L, Ladomersky E, Lauing KL, Wu M, Genet M, Gritsina G, Győrffy B (2017). Infiltrating T cells increase IDO1 expression in glioblastoma and contribute to decreased patient survival. Clin Cancer Res.

[192] Li F, Zhang R, Li S, Liu J (2017). IDO1: an important immunotherapy target in cancer treatment. Int Immunopharmacol.

[193] Zhai L, Spranger S, Binder DC, Gritsina G, Lauing KL, Giles FJ, Wainwright DA (2015). Molecular pathways: targeting IDO1 and other tryptophan dioxygenases for cancer immunotherapy. Clin Cancer Res.

[194] Kim ES (2017). Avelumab: first global approval. Drugs.

[195] Kaufman HL, Russell JS, Hamid O, Bhatia S, Terheyden P, D’Angelo SP, Shih KC (2018). Updated efficacy of avelumab in patients with previously treated metastatic Merkel cell carcinoma after≥ 1 year of follow-up: JAVELIN Merkel 200, a phase 2 clinical trial. J Immunother Cancer.

[196] Powles T, Sridhar SS, Loriot Y, Bellmunt J, Mu XJ, Ching KA, Pu J (2021). Avelumab maintenance in advanced urothelial carcinoma: biomarker analysis of the phase 3 JAVELIN Bladder 100 trial. Nat Med.

[197] Choueiri TK, Larkin J, Oya M, Thistlethwaite F, Martignoni M, Nathan P, Powles T (2018). Preliminary results for avelumab plus axitinib as first-line therapy in patients with advanced clear-cell renal-cell carcinoma (JAVELIN Renal 100): an open-label, dose-finding and dose-expansion, phase 1b trial. Lancet Oncol.

[198] Vaishampayan U, Schöffski P, Ravaud A, Borel C, Peguero J, Chaves J, Morris JC (2019). Avelumab monotherapy as first-line or second-line treatment in patients with metastatic renal cell carcinoma: phase Ib results from the JAVELIN Solid Tumor trial. J Immunother Cancer.

[199] Motzer RJ, Penkov K, Haanen J, Rini B, Albiges L, Campbell MT, Venugopal B (2019). Avelumab plus axitinib versus sunitinib for advanced renal-cell carcinoma. N Engl J Med.

[200] Choueiri T, Larkin J, Pal S, Motzer R, Rini B, Venugopal B, Alekseev B (2021). Efficacy and correlative analyses of avelumab plus axitinib versus sunitinib in sarcomatoid renal cell carcinoma: post hoc analysis of a randomized clinical trial. ESMO open.

[201] Motzer RJ, Robbins PB, Powles T, Albiges L, Haanen JB, Larkin J, Mu XJ (2020). Avelumab plus axitinib versus sunitinib in advanced renal cell carcinoma: biomarker analysis of the phase 3 JAVELIN Renal 101 trial. Nat Med.

[202] Bilen MA, Rini BI, Voss MH, Larkin J, Haanen JB, Albiges L, Pagliaro LC (2021). Association of neutrophil-to-lymphocyte ratio with efficacy of first-line avelumab plus axitinib vs. sunitinib in patients with advanced renal cell carcinoma enrolled in the Phase 3 JAVELIN Renal 101 Trial. Clin Cancer Res.

[203] Guzik K, Tomala M, Muszak D, Konieczny M, Hec A, Błaszkiewicz U, Pustuła M (2019). Development of the inhibitors that target the PD-1/PD-L1 interaction—a brief look at progress on small molecules, peptides and macrocycles. Molecules.

[204] Guo L, Wei R, Lin Y, Kwok HF (2020). Clinical and recent patents applications of PD-1/PD-L1 targeting immunotherapy in cancer treatment-current progress, strategy, and future perspective. Front Immunol.

[205] Skalniak L, Zak KM, Guzik K, Magiera K, Musielak B, Pachota M, Szelazek B (2017). Small-molecule inhibitors of PD-1/PD-L1 immune checkpoint alleviate the PD-L1-induced exhaustion of T-cells. Oncotarget.

[206] Zhan M-M, Hu X-Q, Liu X-X, Ruan B-F, Xu J, Liao C (2016). From monoclonal antibodies to small molecules: the development of inhibitors targeting the PD-1/PD-L1 pathway. Drug Discov Today.

[207] Ganesan A, Ahmed M, Okoye I, Arutyunova E, Babu D, Turnbull WL, Kundu JK (2019). Comprehensive in vitro characterization of PD-L1 small molecule inhibitors. Sci Rep.

[208] Awadasseid A, Wu Y, Zhang W (2021). Advance investigation on synthetic small-molecule inhibitors targeting PD-1/PD-L1 signaling pathway. Life Sci.

[209] Musielak B, Kocik J, Skalniak L, Magiera-Mularz K, Sala D, Czub M, Stec M (2019). CA-170–a potent small-molecule PD-L1 inhibitor or not?. Molecules.

[210] Li C, Shi M, Lin X, Zhang Y, Yu S, Zhou C, Yang N (2021). Novel risk scoring system for immune checkpoint inhibitors treatment in non-small cell lung cancer. Transl Lung Cancer Res.

[211] Albiges L, Hakimi AA, Xie W, McKay RR, Simantov R, Lin X, Lee JL (2016). Body mass index and metastatic renal cell carcinoma: clinical and biological correlations. J Clin Oncol.

[212] Cella D, Grünwald V, Escudier B, Hammers HJ, George S, Nathan P, Grimm M-O (2019). Patient-reported outcomes of patients with advanced renal cell carcinoma treated with nivolumab plus ipilimumab versus sunitinib (CheckMate 214): a randomised, phase 3 trial. Lancet Oncol.

[213] Cella D, Motzer RJ, Suarez C, Blum SI, Ejzykowicz F, Hamilton M, Wallace JF (2022). Patient-reported outcomes with first-line nivolumab plus cabozantinib versus sunitinib in patients with advanced renal cell carcinoma treated in CheckMate 9ER: an open-label, randomised, phase 3 trial. Lancet Oncol.

[214] Courcier J, Dalban C, Laguerre B, Ladoire S, Barthélémy P, Oudard S, Joly F (2021). Primary renal tumour response in patients treated with nivolumab for metastatic renal cell carcinoma: results from the GETUG-AFU 26 NIVOREN trial. Eur Urol.

[215] Weiss SA, Djureinovic D, Jessel S, Krykbaeva I, Zhang L, Jilaveanu L, Ralabate A (2021). A phase I study of APX005M and cabiralizumab with or without nivolumab in patients with melanoma, kidney cancer, or non-small cell lung cancer resistant to anti-PD-1/PD-L1. Clin Cancer Res.

[216] Ficial M, Jegede OA, Sant'Angelo M, Hou Y, Flaifel A, Pignon JC, Braun DA (2021). Expression of T-cell exhaustion molecules and human endogenous retroviruses as predictive biomarkers for response to nivolumab in metastatic clear cell renal cell carcinoma. Clin Cancer Res.

[217] McFarlane JJ, Kochenderfer MD, Olsen MR, Bauer TM, Molina A, Hauke RJ, Reeves JA (2020). Safety and efficacy of nivolumab in patients with advanced clear cell renal cell carcinoma: results from the phase IIIb/IV CheckMate 374 Study. Clin Genitourin Cancer.

[218] Flippot R, Dalban C, Laguerre B, Borchiellini D, Gravis G, Négrier S, Chevreau C (2019). Safety and efficacy of nivolumab in brain metastases from renal cell carcinoma: results of the GETUG-AFU 26 NIVOREN Multicenter Phase II Study. J Clin Oncol.

[219] Tannir NM, Cho DC, Diab A, Sznol M, Bilen MA, Balar AV, Grignani G (2022). Bempegaldesleukin plus nivolumab in first-line renal cell carcinoma: results from the PIVOT-02 study. J Immunother Cancer.

[220] Masini C, Iotti C, De Giorgi U, Bellia RS, Buti S, Salaroli F, Zampiva I (2022). Nivolumab in combination with stereotactic body radiotherapy in pretreated patients with metastatic renal cell carcinoma. Results of the phase II NIVES study. Eur Urol.

[221] Lee CH, Shah AY, Rasco D, Rao A, Taylor MH, Di Simone C, Hsieh JJ (2021). Lenvatinib plus pembrolizumab in patients with either treatment-naive or previously treated metastatic renal cell carcinoma (Study 111/KEYNOTE-146): a phase 1b/2 study. Lancet Oncol.

[222] Dudek AZ, Liu LC, Gupta S, Logan TF, Singer EA, Joshi M, Zakharia YN (2020). Phase Ib/II clinical trial of pembrolizumab with bevacizumab for metastatic renal cell carcinoma: BTCRC-GU14-003. J Clin Oncol.

[223] Martini J-F, Plimack ER, Choueiri TK, McDermott DF, Puzanov I, Fishman MN, Cho DC (2020). Angiogenic and immune-related biomarkers and outcomes following axitinib/pembrolizumab treatment in patients with advanced renal cell carcinoma. Clin Cancer Res.

[224] Siva S, Bressel M, Wood ST, Shaw MG, Loi S, Sandhu SK, Tran B (2022). Stereotactic radiotherapy and short-course pembrolizumab for oligometastatic renal cell carcinoma—the RAPPORT trial. Eur Urol.

[225] Tang B, Yan X, Sheng X, Si L, Cui C, Kong Y, Mao L (2019). Safety and clinical activity with an anti-PD-1 antibody JS001 in advanced melanoma or urologic cancer patients. J Hematol Oncol.

[226] Pal SK, McGregor B, Suárez C, Tsao CK, Kelly W, Vaishampayan U, Pagliaro L (2021). Cabozantinib in combination with atezolizumab for advanced renal cell carcinoma: results from the COSMIC-021 Study. J Clin Oncol.

[227] Powles T, Atkins MB, Escudier B, Motzer RJ, Rini BI, Fong L, Joseph RW (2021). Efficacy and safety of atezolizumab plus bevacizumab following disease progression on atezolizumab or sunitinib monotherapy in patients with metastatic renal cell carcinoma in IMmotion150: a randomized phase 2 clinical trial. Eur Urol.

[228] Jung KH, LoRusso P, Burris H, Gordon M, Bang YJ, Hellmann MD, Cervantes A (2019). Phase I Study of the Indoleamine 2,3-Dioxygenase 1 (IDO1) Inhibitor Navoximod (GDC-0919) Administered with PD-L1 Inhibitor (Atezolizumab) in Advanced Solid Tumors. Clin Cancer Res.

[229] Choueiri TK, Motzer RJ, Rini BI, Haanen J, Campbell MT, Venugopal B, Kollmannsberger C (2020). Updated efficacy results from the JAVELIN Renal 101 trial: first-line avelumab plus axitinib versus sunitinib in patients with advanced renal cell carcinoma. Ann Oncol.

[230] Fong L, Hotson A, Powderly JD, Sznol M, Heist RS, Choueiri TK, George S (2020). Adenosine 2A receptor blockade as an immunotherapy for treatment-refractory renal cell cancer. Cancer Discov.

